# Immune-Proteome Profiling in Classical Hodgkin Lymphoma Tumor Diagnostic Tissue

**DOI:** 10.3390/cancers14010009

**Published:** 2021-12-21

**Authors:** Alex Reza Gholiha, Peter Hollander, Liza Löf, Anders Larsson, Jamileh Hashemi, Johan Mattsson Ulfstedt, Daniel Molin, Rose-Marie Amini, Eva Freyhult, Masood Kamali-Moghaddam, Gunilla Enblad

**Affiliations:** 1 Experimental and Clinical Oncology, Department of Immunology, Genetics and Pathology, Uppsala University, SE-752 36 Uppsala, Sweden; jamileh.hashemi@igp.uu.se (J.H.); johan.mattsson-ulfstedt@igp.uu.se (J.M.U.); daniel.molin@igp.uu.se (D.M.); gunilla.enblad@igp.uu.se (G.E.); 2 Clinical and Experimental Pathology, Department of Immunology, Genetics and Pathology, Uppsala University, SE-752 36 Uppsala, Sweden; peter.hollander@igp.uu.se (P.H.); rose-marie.amini@igp.uu.se (R.-M.A.); 3 Department of Immunology, Genetics & Pathology, Science for Life Laboratory, Uppsala University, SE-751 08 Uppsala, Sweden; liza.lof@igp.uu.se (L.L.); masood.kamali@igp.uu.se (M.K.-M.); 4 Department of Medical Sciences, Clinical Chemistry, Uppsala University, SE-751 85 Uppsala, Sweden; anders.larsson@akademiska.se; 5 Department of Cell and Molecular Biology, National Bioinformatics Infrastructure Sweden, Science for Life Laboratory, Uppsala University, SE-752 36 Uppsala, Sweden; eva.freyhult@medsci.uu.se

**Keywords:** Hodgkin lymphoma, proteomics, proximity assays, tumor microenvironment, PD-L1, LAG3 CCL17, biomarkers, Immunology

## Abstract

**Simple Summary:**

The proximity extension assay (PEA) method enables the detection of proteins in tissue lysates and plasma with high specificity and sensitivity. Knowledge regarding the immune proteome profile in classical Hodgkin Lymphoma (cHL) tumor microenvironment (TME) is critical in an era of emerging immunotherapies and precision medicine. This study identifies several important immune markers that distinguish cHL tissue from reactive lymph nodes and introduces new potential therapeutic targets in an era of personalized medicine.

**Abstract:**

In classical Hodgkin Lymphoma (cHL), immunoediting via protein signaling is key to evading tumor surveillance. We aimed to identify immune-related proteins that distinguish diagnostic cHL tissues (=diagnostic tumor lysates, *n* = 27) from control tissues (reactive lymph node lysates, *n* = 30). Further, we correlated our findings with the proteome plasma profile between cHL patients (*n* = 26) and healthy controls (*n* = 27). We used the proximity extension assay (PEA) with the OlinkTM multiplex Immuno-Oncology panel, consisting of 92 proteins. Univariate, multivariate-adjusted analysis and Benjamini–Hochberg’s false discovery testing (=Padj) were performed to detect significant discrepancies. Proteins distinguishing cHL cases from controls were more numerous in plasma (30 proteins) than tissue (17 proteins), all Padj < 0.05. Eight of the identified proteins in cHL tissue (PD-L1, IL-6, CCL17, CCL3, IL-13, MMP12, TNFRS4, and LAG3) were elevated in both cHL tissues and cHL plasma compared with control samples. Six proteins distinguishing cHL tissues from controls tissues were significantly correlated to PD-L1 expression in cHL tissue (IL-6, MCP-2, CCL3, CCL4, GZMB, and IFN-gamma, all *p* ≤0.05). In conclusion, this study introduces a distinguishing proteomic profile in cHL tissue and potential immune-related markers of pathophysiological relevance.

## 1. Introduction

In the tumor microenvironment (TME) of classical Hodgkin lymphoma (cHL), sparsely distributed Hodgkin and Reed–Sternberg (HRS) cells are surrounded by an abundant number of leukocytes and stromal cells [1,2,3]. Immunoediting of the TME by HRS cells is vital for escaping tumor immune surveillance [3]. With best-practice treatments, still a significant proportion of patients with cHL relapse with subsequently poor prognosis [4]. In an era of personalized medicine, tailored therapeutic management of malignant disease is highly biomarker-driven [5]. While gene expression profiling and mRNA quantification studies in cHL have been reported [6,7,8,9,10], high throughput proteome studies in cHL are restricted and based on commercial HRS cell lines [11,12,13,14]. Hence, there is an unmet need to explore the proteome profile of cHL to identify key biomarkers and new potential therapeutic targets in an era of precision medicine [15,16].

Methods for characterizing cancer proteome are rapidly evolving within cancer research [17]. The proximity extension assay (PEA) enables high throughput detection and quantification of a large number of biomarkers with high sensitivity and specificity [18,19]. The PEA technology has been widely used to analyze various body fluids including tissue lysates and plasma [18,19]. The technology is believed to complement mass spectrophotometry (MS) by a higher ability for sensitive detection of low abundance proteins in minute sample volumes [20].

To deepen our knowledge of key components of the proteome profile in cHL, this study aimed to identify distinguishing immune-related proteins in cHL tissues compared with reactive lymph nodes and their association to plasma proteome profile in cHL.

## 2. Materials and Methods

### 2.1. Study Cohort and Study Samples

**Figure 1:**
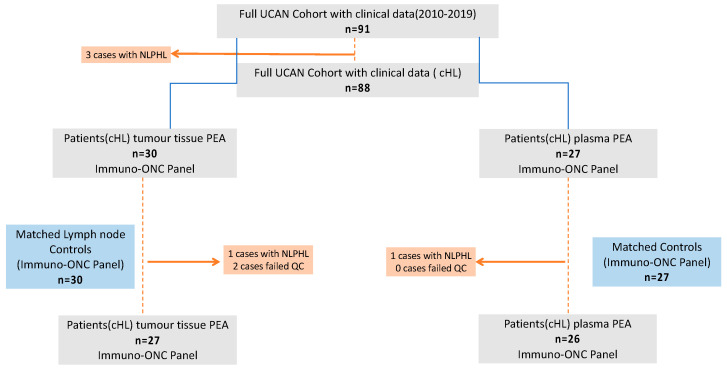
Flowchart of patients included in the current study: UCAN = Uppsala Umeå Comprehensive Cancer Consortium biobank program, cHL = classical Hodgkin lymphoma, NLPHL = nodular lymphocyte-predominant Hodgkin lymphoma, QC = quality control, PEA = proximity extension assay, and Immuno-ONC = Immuno-Oncology.

This study included patients from the biobank program U-CAN (Uppsala Umeå Comprehensive Cancer Consortium). The U-CAN program has, since 2010, collected data and created a biobank with blood and tissue samples with various cancer diagnoses including lymphoma. The current cohort consisted of 88 patients included in U-CAN and was confined to cHL patients with available plasma samples and frozen diagnostic biopsies diagnosed between 2010 and 2019 (*n* = 27). One patient was included at relapse with a first diagnosis in 1989 (Figure 1). As controls, 30 healthy study subjects with lymph nodes classified histopathologically as reactive lymphadenopathy were used. The controls were matched for age and gender.

The diagnostic biopsies for patients with cHL (*n* = 27) were obtained from lymph nodes in the axilla (*n* = 2), neck (*n* = 21), and mediastinum (*n* = 2). Two patients had missing information on lymph node location. Biopsy location from non-malignant lymph nodes (*n* = 30) were neck (*n* = 18), groin (*n* = 5), axilla (*n* = 5), and mesenterium (*n* = 1). One patient had missing data on lymph node location. Of the cHL plasma samples (*n* = 26), 25 were from the same patients as the tissue samples from cHL patients. Plasma control samples (*n* = 27) were obtained after consent and matched for age and gender as the tissue controls. Clinical data were obtained from patient hospital records. Stage of disease was defined according to the Ann Arbor classification, and advanced stage was defined as IIB-IVB [21,22]. Analysis of EBV infection in HRS cells was performed using immunohistochemistry (IHC) for latent membrane protein 1 (LMP1) and in situ hybridization (ISH) for Epstein–Barr virus (EBV)-encoded small RNAs (EBERs).

### 2.2. Tissue Lysates Preparation and Plasma Samples

The tissue samples were snap-frozen in liquid nitrogen and stored at −80 °C after surgery. Tissue samples were cut into thin slices and were lysed as previously described [23]. Tissue lysate preparation was performed at Uppsala Biomedical Center (BMC), Uppsala University, SciLifeLab. Tumor tissues and control lymph node tissues were mixed with 2 mm in diameter zirconium beads (Next Advance Inc., Troy, NY, USA) and lysis buffer (50 mM Tris-HCl, pH 7.4, 150 mM NaCl, 1 mM EDTA, pH 8, 1% Triton X-100, 0.1% sodium deoxycholate, a protease inhibitor (Roche Complete Mini, Mannheim, Germany). The ratio of sliced tissue mass: volume of lysis buffer: zirconium beads weight was 1:4:2. The tissues were homogenized using Bullet Blender (BBX24B-CE, Next Advance, Inc.) according to the manufacturer’s recommendation for lymphatic tissue. The mixtures were then centrifuged at 13,000 rpm for 10 min at 4 °C, and the supernatants were transferred to new tubes. The total protein concentration of tissue lysates was measured using PierceTM BCA Protein Assay kit (ThermoFisher, Rockford, IL, USA).

To determine the optimal buffer condition and total protein concentrations for PEA analysis, one tumor and one healthy lymph node sample were lysed, and total protein concentrations were measured. For each sample, three different dilutions of 0.5, 0.125, and 0.031 µg/µL total protein concentrations were prepared in PEA dilution buffer (Olink, Uppsala, Sweden) and analyzed with multiplex PEA, using Immuno-Oncology panel (Olink Proteomics™, Uppsala, Sweden), (Table S1), according to the manufacturer’s instructions and as described by Shen et al. [24]. The optimal total protein concentration was determined to 0.125 μg/μL. For the plasma samples, 1 µL of sample was analyzed using the same Immuno-Oncology panel.

### 2.3. Proximity Extension Assay Overview, Plate Distribution, and Data Output

PEA relies on dual antigen recognition via matched antibodies linked with a unique DNA oligonucleotide that undergoes hybridization upon target recognition. In this way, the PEA method reduces antibody cross-reactivity similar to the proximity ligation assay (PLA) [25]. The hybridized DNA oligonucleotides are subsequently subjected to enzymatic DNA extension and real-time PCR (qPCR) to quantify the DNA amplicons [18]. The PEA steps include various internal controls and monitoring steps and allows quantification of 92 proteins and four controls in femtomolar range, using only one microliter of biofluids [18,24]. The workflow for normalization, quality controls, and the list of proteins included in the panel are available elsewhere [26].

In the current study, tissue lysates and plasma samples were randomized within the same but separate plates prior to the PEA analysis. Normalized Protein eXpression (NPX) is a log2 arbitrary scale unit that was used as data outcome. One NPX difference corresponds to a two-fold change in the concentration of the protein in the analyzed biosample. NPX is based on the cycle threshold (Ct) value that corresponds to the total of PCR cycles required for the fluorescent signal to surpass background levels for each protein, and normalization algorithms are based on a so-called inter-plate controls (IPCs) described in detail elsewhere [18]. In PEA, the lower limit of detection (LOD) is determined by comparing protein NPX with so-called “background” NPX values calculated via linear regression and different algorithms [18]. Data values with levels under LOD were replaced with a fixed value (=LOD).

### 2.4. Statistics

R version 3.3.3.vas used for all analyses. Missing data were replaced with mean imputation for 4 of 2392 data point entries for plasma PEA analysis. Principal component analysis (PCA) was used for dimension reduction and review of the associations between controls and cHL cases. Comparison between a continuous variable and two groups was analyzed using the two-sample Welch *t*-test and Wilcoxon rank–sum test. Adjustment for age and sex was further conducted with multivariate linear regression and propensity score matching, caliper = 0.2. Clinicopathological correlations were calculated based on NPX values of cHL tissues. Pearson correlation analysis with correlation coefficient (=r) and associated *p*-value was used to estimate the correlation between two continuous variables. Receiver operating curves (ROC) with associated area under curve (AUC) were used to estimate variable predictive values for cHL cases compared with controls. Benjamini-Hochberg’s false discovery rate method was used for adjusting for multiple testing (=Padj). All proteins (*n* = 92), including those below LOD frequency, were accounted for, when adjusting for multiple testing. Padj-values < 0.05 were considered significant.

## 3. Results

Patient characteristics for the analyzed cohort are shown in Table 1, with comparable demographics compared with the original cohort. All proteins and their abbreviations are described in Table S1. In tissues, proteins with a high frequency of values below LOD was IL-1 alpha (96%), ADGRG1 (95%), MUC-16 (81%), KIR3DL1 (79%), IL-10 (82%), TNFRSF12A (91%), ARG1 (88%), and CXCL12 (93%). The distribution of values <LOD frequency was evenly distributed between cHL tissues and control tissues in these cases (see Table S2). Since all values <LOD were replaced with LOD they automatically did not generate any significant differences between cHL and control cases when frequent values of <LOD were observed for both cHL tissues and control tissues.

### 3.1. Proteins Distinguishing Patients from Controls

**Figure 2:**
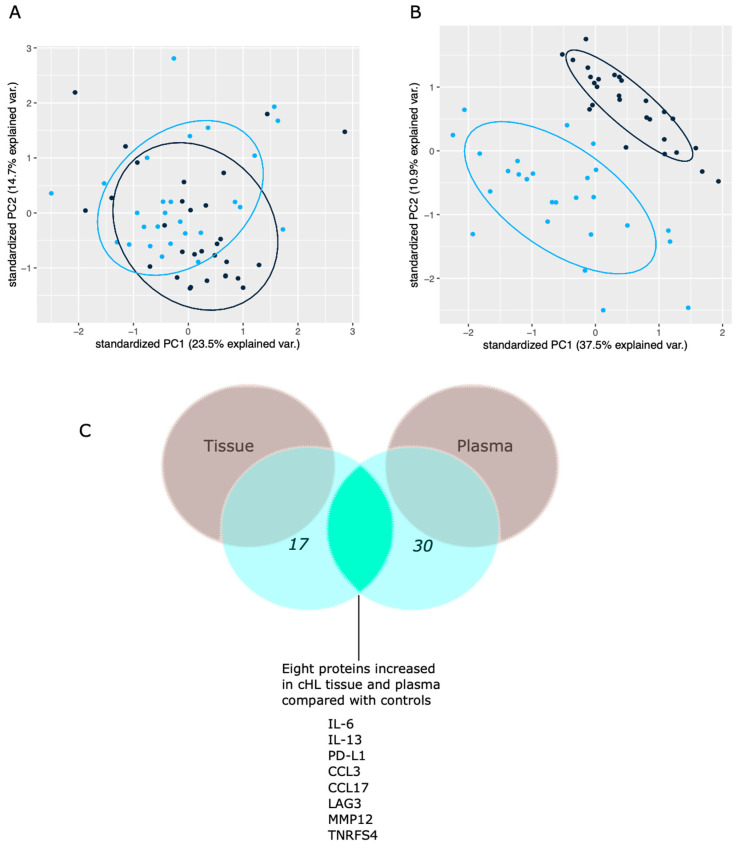
Principal component analysis (PCA) and Venn diagram for the proteome profile: (**A**) PCA plot for tissue. Dots demonstrate individual sample distribution of 27 cHL tumor tissues analyzed with PEA (blue) and corresponding control (black) lymph nodes, *n* = 30. (**B**) PCA plots for plasma. Dots demonstrate individual sample distribution of 26 cHL patients (blue) and controls (black), *n* = 27; a total of 92 proteins were included. PC = principal component. (**C**) Schematic Venn diagram showing the number of proteins with significantly higher levels in cHL cases than controls in both tissue and plasma after univariate analysis and adjusted analysis.

After multivariate tests adjusting for age and gender, PS matched comparison, and for adjusting for multiple testing, 17 out of 92 proteins levels (Table 2) were identified to be significantly different in cHL tissues compared with control tissues (all Padj < 0.05). All 17 proteins showed high predictive accuracy (AUC between 0.82–0.92) to differentiate between cHL cases and controls. TIE2 and IL7 were significantly decreased in cHL tissues, while all other proteins (*n* = 14) were increased in cHL tissues compared with control tissues. PCA plot overview of the tissue protein pattern showed a modest separation between cHL cases and control study subjects (Figure 2A).

The biological function annotation based on bioinformatics databases (Uniprot, Human Protein Atlas, Gene Ontology (GO) and DisGeNET) of the 17 identified proteins were in the category of Chemotaxis/Inflammation (IL-6, IL-13, MCP1, MCP2, MCP4, CCL3, CCL4, CCL17, IL-7, and IFN-gamma), and immune-suppression/promotion (PD-L1, LAG-3, CD70, and TNFRSF4), apoptosis (Granzyme B), and extracellular matrix remodeling (MMP12, TIE2).

Of all the identified 17 proteins, eight proteins (PD-L1, IL-6, CCL17, CCL3, IL-13, MMP12, TNFRS4, and LAG3) were also significantly increased in plasma samples from cHL patients compared with plasma from controls (Figure 2C). The obtained results and biological annotation for all 17 proteins are summarized in Table 3. The discrepancy between cHL patients and controls proteome profiles in plasma was apparent, with 30 out of 92 proteins identified to be expressed significantly differently in cHL patients than controls (Table S3). In addition, PCA plot overview of the protein pattern in plasma showed an apparent separation between cHL cases and controls (Figure 2B).

### 3.2. Clinicopathological Correlations

The cohort was too small to generate significant correlations between protein NPX levels in patient tissues and clinical features after multiple testing (all Padj > 0.05), but the unadjusted *p*-values are presented in Table S4. CCL17 NPX levels in cHL tissues were higher in cHL nodular sclerosis subtype cases (*p* = 0.029). In cHL tissue, higher PD-L1 NPX levels correlated with EBV+ cases (*p* = 0.020) and male sex (*p* = 0.005). CCL3 NPX levels in cHL tissues were higher in EBV+ cases (*p* = 0.011). Unadjusted for multiple testing, correlations between proteins distinguishing cHL tissues are available in Table S5. PD-L1 NPX levels in cHL tissues correlated with NPX tissue levels of; CCL3 (r = 0.79 (*p* < 0.001)), CCL4 (r = 0.82 (*p* < 0.001)), IFN-gamma (r = 0.63 (*p* < 0.001)), MCP-2 (r = 0.67 (*p* < 0.001)), IL-6 (r = 0.38 (*p* = 0.05)), and GZMB (r = 0.061 (*p* = 0.001)).

## 4. Discussion

By implementing PEA analysis on tumor tissues and plasma, we identified 17 proteins in cHL tissues and 30 proteins in plasma, significantly distinguishing cHL cases from controls. Eight (IL-6, PD-L1, CCL17, MMP12, TNFRSF4, CCL3, IL-13, and LAG3) of the identified 17 proteins in cHL tissues were also significantly elevated in cHL plasma compared with controls. In addition, six of the 17 distinguishing proteins in cHL tissue were often positively correlated to PD-L1 expression in cHL tissue (IL-6, MCP-2, CCL3, CCL4, GZMB, and IFN-gamma).

Reports reviewing and investigating gene expression profiling and mRNA quantification studies in cHL [6,7,8,9,10] and high throughput proteome studies in cHL [11,12,13,14] will be discussed next in regard to overlaps and similarities between our markers individually for each protein, focusing on our findings in cHL tissues.

### 4.1. Immunobiomarkers Elevated in cHL Tissues and Plasma Samples (n = 8)

IL-6 is a pluripotent cytokine produced by macrophages and lymphocytes with pro-tumorigenic signaling abilities across different malignancies [48]. In cHL, IL-6 can be found in various cells, including HRS cells confirmed by IHC and ISH methods [8,30]. We have previously reported that the presence of >1% of IL-6 + non-malignant cells in the tumor microenvironment is associated with poor prognosis in cHL [30], hence aligned with current study result, identifying IL-6 as a distinguishing cHL protein increased in cHL tissues. The weak correlation between PD-L1 and IL-6 observed in the current study is further aligned with studies reporting that IL-6 can induce PD-L1 expression in tumor cells and immune cells [30,49,50].

CCL17 (also known as TARC) is produced by macrophages (M2 type), and dendritic cells in the TME, and is mainly believed to recruit CCL4+ regulatory T-cells, promoting an anergic TME [51]. CCL17 is a well-known biomarker in cHL and elevated in serum of pretreated cHL patients [52], with predominance in patients with nodular sclerosis [53]. In cHL, CCL17 is expressed by HRS cells [7,8,14]. Our findings show an increase in cHL tissue predominantly in patients with nodular sclerosis and a significant increase in plasma, congruent with previous studies [53] of CCL17 in cHL, supporting the central role of CCL17 in the pathophysiology of cHL.

PD-L1 is a vital checkpoint ligand in several malignancies, with extensive evidence regarding its expression on HRS cells and surrounding leukocytes in the TME of cHL [40,41]. Hence a critical finding in the current study was the increased PD-L1 levels in cHL tissues compared with control tissues. We did not observe any significant differences for other PD-L1 related proteins (PD-L2 and PDCD1) in plasma or tissues between cHL cases and controls. However, PD-L1 levels correlated with several identified proteins increased in cHL tissues, CCL4, CCL3, MCP-2, IL-6, GXMB, and IFN-gamma. Further, higher PD-L1 expression was observed in EBV+ cases, congruent with studies indicating that EBV can drive increased expression of PD-L1 in cHL via complex mechanisms based on EBV-associated proteins like LMP-1 [54,55]. The PD-L1 correlation with male sex found in the current study has also been seen in non-small cell lung cancer but, to our knowledge, lacks biological explanation [56].

LAG3 (Lymphocyte-activation gene 3) is a cell surface protein expressed on multiple immune cells, including macrophages and T cells [57]. LAG3 is a critical checkpoint inhibitor in the TME, described in various cancer forms, including cHL, with ongoing clinical trials (ClinicalTrials.gov identifier: NCT02061761). IHC studies show that HRS cells rarely express LAG-3 (5.2% of the cases). However, LAG-3+ leukocytes are frequently found in the proximity of HRS cells [27,28]. In addition, a study by Abro et al. [29] found that levels of LAG3 mRNA were 5–10-fold higher in cHL tissues compared with control tissues and correlated with infiltration of CD4+-, CD8 +T cells and macrophages. In the current study, we found an increase of LAG3 cHL tissues and plasma compared with controls, aligned with previous evidence [29].

CCL3 (also known as MIP-1 alpha) is a protein with inflammatory properties that recruit immunosuppressive phenotypes of macrophages to the TME [58,59]. In cHL, CCL3 mRNA is increased in cHL tissues compared with control tissues, especially in EBV + cases [9]. Further, CCL3 is upregulated in tumor-associated macrophages (TAMs) [35]. In the current study, we found increased levels of CCL3 in both cHL tissues and plasma compared with tissues and plasma from controls. Moreover, EBV+ cases had higher tissue CCL3 levels in line with previous findings elsewhere [9]. Furthermore, we observed a strong linear correlation between PD-L1 levels and CCL3, which has previously been observed in other malignancies [60,61]. This may reflect a phenotypical subpopulation in the TME of CCL3+, PD-L1+ immune cells or indicate that CCL3 mainly recruits immunosuppressive PD-L1+ immune cells in the TME of cHL.

In the current study, IL-13, TNFSF4, and MMP12 were also observed to be elevated in both cHL tissues and plasma compared with controls. However, studies on these proteins are limited in cancer and particularly cHL. IL-13 is elevated in HRS cells [32,33] and hypothesized to act as an autocrine growth factor for HRS cells [31,34]. TNFSF4 (also known as OX40L) is elevated and expressed by subtypes of T-helper cells in the TME of cHL [37,38], but HRS lack expression [39]. MMP12, (also known as macrophage metalloelastase, MME) is mainly involved in tissues remodeling and extracellular matrix organization and is primarily produced by macrophages but lacks studies in cHL [62].

### 4.2. Proteins with Decreased levels in cHL Tissues (n = 2)

TIE2 (also known as angiopoietin-1 receptor) was decreased in cHL tissues compared with control tissues. TIE2 interacts with ANPT1 and partially with ANPGT2 [63,64]. ANGPT2-TIE2 activation increases endothelial permeability and angiogenesis, favoring tumor progression, while ANGPT-1-TIE2 maintains membrane permeability and quiescent vascular remodeling affecting tumor survival negatively [63,64]. Moreover, even if not significant in present study, we observed that ANGPT1 was decreased in cHL tissues, while ANGPT2 was increased (Table S2). To our knowledge, there is a lack of reports regarding TIE-2 expression in cHL tissues.

IL-7, which is known to be produced by stromal cells as well as dendritic cells, plays an essential role in the early stages of B-cell maturation [65] and T-cell maturation [66]. In cHL, IL-7 gene transcripts and proteins are elevated in HRS cells and in cHL patient plasma [43,44] postulated to act as an autocrine growth factors. However, IL-7 can promote survival of effector and memory T-cells [67], hence the role of IL-7 in cHL remains to be determined.

### 4.3. Immunobiomarkers Elevated in cHL Tissues but Not Plasma (n = 7)

CCL4 (also known as macrophage inflammatory protein beta (MIP-1 beta)) is a secretory protein with chemokine and inflammatory functions. Studies investigating CCL4 expression in cHL are limited [68]. CCL4 lacks expression in HRS cells but is upregulated in TAMs found in cHL [35]. We observed a strong correlation between CCL4 and PD-L1 levels, which may indicate that CCL4 plays an important role in recruiting PD-L1+ immune cells.

IFN-gamma is an inflammatory protein attributed to possessing antitumor effects like recruiting effector T-cells [69]. However, effects such as modulating STAT signaling systems and suppressing effector T-cells in the TME are also described, favoring tumor progression [69]. Elevated levels of IFN-gamma in cHL have been reported using various methods [8,9,36]. We observed that cHL tissues levels were higher in EBV+ cases. The association between IFN-gamma and EBV+ cases remains unexplained but has been seen in other malignancies as well [70]. Reports that IFN-gamma may induce PD-L1 expression are widely emerging in different malignancies and seen in various tumor cell lines and immune cells [49,71,72]. This is congruent with our observed correlation between IFN-gamma levels and PD-L1 in cHL. MCP1 (also known as CCL2) is a monocyte-attracting cytokine in cancer associated with TAMs [73]. The presence of MCP1 genome has been observed in cHL [42]; however, IHC studies are limited. MCP-2 (also known as CCL8) stimulates chemotactic activity for various immune cells. In cHL, MCP2 gene expression is upregulated in HRS cells [6]. In the current study, we observed an association between MCP2 and PD-L1 levels in cHL tissues suggesting that MCP-2 recruits PD-L1 immune cells. MCP-4 (also known as CCL13) is involved in several biological processes such as chemotactic activity for T-cells, monocytes, and eosinophils [74]. In cHL, MCP-4 is expressed by macrophages, dendritic cells, and HRS cells [7]. There are currently limited studies on MCP4 roles in cancer and particularly in cHL. GZMB [47] and CD70 [45,46] were further two proteins identified distinguishing cHL tissue from controls, their pathophysiological role in cHL is unknown and warrants further investigation.

### 4.4. General Comments Regarding the Identified Proteome Profile

Since plasma protein patterns are more prone to be affected by multiple organs and are unclear in their implication in the TME, the focus of this study has been centralized regarding the proteome profile in cHL tissue. However, the study failed to identify an apparent correlation of why some proteins were expressed differently in both tissue and plasma while others were not (see Table 3).

However, the discrepancy in the numbers of identified proteins in plasma compared with tissue could be explained by the tissue controls, which were lymph nodes diagnosed with reactive lymphadenopathy. A reactive inflammation in these lymph nodes may increase several inflammatory proteins that overlap with the pathophysiology in the TME of malignancies and contribute to the reduced differences in protein levels compared with cHL tissue. This particularly resonates with the fact that most pre-selected proteins in the current study have known functions in the natural immune response. In addition, plasma protein levels could be affected by other sources in the body, as discussed above, and hence not completely reflect the protein composition of the tissues.

Most of the identified proteins in the current study have been studied across different malignancies and to a certain degree in cHL, but we also identified several markers with limited studies in cHL (MMP12, CD70, IL-7, MCP-4, TIE2, IL-13, TNFSF4, and GZMB, Table 3). In addition, several proteins identified distinguishing cHL tissues from control tissues are known to be associated with macrophages in either their source of production or ability to recruit macrophages (MCP-1, MCP-2, MCP-4, IL-6, MMP12, CCL3, and CCL4). This could be expected since macrophages are a source for chemokines and cytokines, and several studies reports an adverse prognostic outcome for high proportions of TAMs in the TME of cHL [75,76,77]. Moreover, six proteins (CCL3, MCP-2, IFN-gamma, Granzyme B, IL-6, and CCL-4) were correlated with PD-L1 levels in cHL tissues, which is congruent with the extensive evidence regarding the role of PD-L1 in cHL [40,41].

### 4.5. Study Limitations and Strengths

Most of the 17 identified distinguishing proteins in cHL tissues have been investigated to different degrees in cHL, while others have been studied to a lesser extent (see Table 3). Adding additional IHC analysis to the current study would help re-validate many of these proteins and shed light on their spatial distribution in the TME. Another limitation is that we used lymph nodes diagnosed with reactive lymphadenopathy, hence similar biological mechanisms could be involved in reactive lymph node and malignant processes, and as mentioned before, would explain why plasma discrepancies between cHL and controls were more apparent (30/92) compared with tissues (17/92). On the other hand, this could also be a strength since it would identify the most specific biomarkers for malignant processes compared with benign inflammation processes. However, it would be of additional value to include healthy lymph nodes.

Moreover, the limited number of patient biofluids, *n* = 27 (tissues) and *n* = 26 (plasma) make the study results vulnerable. The unique character of this study is the usage of multiplex PEA, with several advantages as described previously [21], including its accuracy to detect low abundance proteins. Moreover, a strength of the study is the usage of reactive lymph nodes as controls. The handling of values below LOD can be addressed in different ways, replacing values below LOD with LOD, LOD/SQRT2, or using actual data, even though below LOD [78]. All three methods were used in the current study (data only shown for the first method) and did not show any difference in the outcome of study results.

## 5. Conclusions

We found 17 proteins that separate cHL tissues from control tissues, eght of these proteins (PD-L1, IL-6, CCL17, IL-13, CCL3, TFNSF4, MMP12, and LAG3) were also increased in plasma. In addition, several identified immune biomarkers (IL-6, MCP-2, CCL3, CCL4, GZMB, and IFN-gamma) correlated with PD-L1 levels in cHL tissues. Our results thus deepen our insights regarding proteome profile of immune-markers in cHL and introduce new potential targets in an era of personalized medicine.

## Figures and Tables

**Table 1:** Demographics.

	Entire Cohort (cHL) 2010–2019 *n* = 88	Patient Plasma PEA *n* = 26	Control: Plasma PEA *n* = 27	Patient: Tissue PEA *n* = 27	Controls: Tissue PEA *n* = 30
Age (y): Median (Range)	41 (12–85)	44 (21–85)	45 (20–78)	45 (21–85)	45.50 (22–83)
Age ≥ 60 (*n*)	23 (26%)	7 (27%)	8 (30%)	8 (30%)	7 (23%)
Male Sex (*n*)	58 (66%)	17 (65%)	18 (67%)	18 (67%)	21 (70%)
Follow-up time (y); Median (range)	4.50 (0.36–26.00)	4.75 (0.66–9.26)	NA	4.77 (0.66–26.00)	NA
5 year OS probability	85%	87%	NA	88%	NA
2 year OS probability	91%	92%	NA	93%	NA
2 year EFS probability	84%	81%	NA	82%	NA
Advanced stage (*n*) (IIB-IVA)	56 (64%)	12 (46%)	NA	12 (57%)	NA
WHO 0–1 (*n*)	74 (95%) Missing = 10	24 (92%)	NA	25 (100%) Missing = 2	NA
IPS ≥ 2 (*n*)	44 (56%) Missing = 10	17 (68%) Missing = 1	NA	11 (49) Missing = 4	NA
Treated with BEACOPP at first-line (*n*)	14 (16%) Missing = 1	*n* = 4 (15%)	NA	4 (15%) Missing = 1	NA
Treated with ABVD at first-line (*n*)	55 (63%) Missing = 1	*n* = 18 (69%)	NA	17 (65%) Missing = 1	NA

ABVD (doxorubicin, bleomycin, vinblastine, and dacarbazine); BEACOPP (bleomycin, etoposide, doxorubicin, cyclophosphamide, vincristine, procarbazine, and prednisone); IPS = international prognostic index; EFS = event-free survival; OS = overall survival; WHO, performance status according to the World Health Organization (ECOG); Advanced stage = according to Ann Arbor; NA = not available.

**Table 2:** Proteins with significant differences in cHL tumor tissue versus control tissues.

	Univariate			Multivariate	Predictive	
	Mean Difference. NPX	*p*	Padj	Padj	AUC	Pw (adj)
TIE2	−0.645	<0.001	<0.001	<0.001	0.870	<0.001
IL7	−0.585	<0.001	0.001	0.002	0.833	<0.001
IL6	2.879	<0.001	<0.001	<0.001	0.922	<0.001
MCP-1	0.985	<0.001	0.001	0.002	0.825	0.001
MCP-4	2.089	<0.001	0.006	0.002	0.821	0.001
MCP-2	1.688	<0.001	0.001	0.001	0.823	0.001
CCL4	1.728	<0.001	<0.001	<0.001	0.878	<0.001
PD-L1	1.094	<0.001	0.001	<0.001	0.854	<0.001
CD70	0.799	<0.001	0.003	0.001	0.799	0.005
CCL3	1.291	<0.001	0.003	0.001	0.805	0.003
TNFRSF4	1.260	<0.001	<0.001	<0.001	0.835	<0.001
CCL17	3.800	<0.001	<0.001	<0.001	0.917	<0.001
IFN-gamma	2.363	<0.001	<0.001	<0.001	0.893	<0.001
MMP12	1.891	<0.001	<0.001	0.007	0.817	0.001
LAG3	2.119	<0.001	<0.001	<0.001	0.925	<0.001
IL13	2.382	<0.001	<0.001	<0.001	0.909	<0.001
GZMB	0.952	0.001	0.049	0.042	0.762	0.040

Comparing 29 cHL cases with 30 controls. Mean normalized protein expression (NPX) difference = mean NPX in the cHL patients minus mean in the control group. One NPX in log2 difference corresponded to a two-fold difference in protein concentration in the tissue. In univariate analysis: *P* = *p*-value was calculated with two-sample Welch test; Padj = FDR-adjusted values corrected for multiple testing (all 92 proteins). In multivariate analysis = linear regression adjusting for age and gender. AUC (area under curve) = accuracy for predicting between cHL cases and controls. Pw (adjust) = *p*-value retrieved with Wilcoxon test and adjusted for multiple testing (FDR); CCL = C–C motif chemokine ligand; PD-L = programmed death ligand; IFN = Interferon; IL = Interleukin; MCP = monocyte chemotactic proteins; LAG = lymphocyte activating gene; Chl = classical Hodgkin lymphoma; GZMB = Granzyme-B; CD = cluster of differentiation; TIE2 = tyrosine-protein kinase receptor Tie-1, also called angiopoietin receptor 1; TNFRSF4 = TNF Receptor Superfamily Member 4; MMP12 = Matrix Metallopeptidase 12.

**Table 3:** Summary of 17 proteins that distinguished cHL tissues from control tissues.

Protein	Tissue	Plasma	Biological Annotation *	Cellular Annotation *	Studies in cHL
*LAG3*	Increased in cHL cases	Increased in cHL cases	Suppressed tumor immunity:	Membrane	Mainly upregulated in Tregs adjacent to HRS cells but also observed in macrophages [27,28,29]
*CCL17*	Increased in cHL cases	Increase in cHL cases	Chemotaxis: Produced by several leukocytes including M2 macrophages	Secretory	Confirmed in HRS cells, and monocytes in the TME [7,8,14]
*IL6*	Increased in cHL cases	Increase in cHL cases	Inflammation/cell Survival signaling. Produced by several leukocytes including macrophages	Secretory	Confirmed in HRS cells, and various leukocytes [8,30]
*IL13*	Increased in cHL cases	Increased in cHL cases	Inflammation/cell survival signaling	Secretory	Confirmed in HRS cells, and various lymphocytes [31,32,33,34]
*CCL4*	Increased in cHL cases	Non-significantly increase in cHL cases	Chemotaxis. Recruit Tregs	Secretory	Higher levels in TAMs, HRS cells mainly negative [35]
*IFN-gamma*	Increased in cHL cases	Non-significantly increase in cHL	Inflammation, cell survival signaling	Secretory	Confirmed in HRS cells [8,9,36]
*TIE2*	Decreased in cHL	Non-significantly decreased in cHL	Vascular remolding. Migration/permeability	Membrane. and secretory	No data available
*TNFRSF4*	Increased in cHL cases	Increased in cHL cases	Chemotaxis/Cell signal survival. Induced host antitumor immunity	Membrane and intracellular	Confirmed in T-cells in cHL. Status in HRS cells limited [37,38,39]
*PD-L1*	Increased in cHL cases	Increased in cHL cases	Suppressed host tumor immunity	Cell membrane	Confirmed in HRS cells and surrounding leukocytes [40,41]
*MCP-1*	Increased in cHL cases	Non--significantly increase in cHL	Chemotaxis for monocytes	Secreted	Confirmed in Monocytes and HRS cells [42]
*MCP-2*	Increased in cHL cases	Non-significantly increase in cHL	Chemotaxis for various leukocytes	Secreted	Confirmed in HRS cells [6]
*IL7*	Decreased in cHL	Increased in cHL	Promoted immune host response	Secreted	Confirmed in HRS cells [43,44]
*CD70*	Increased in cHL cases	Non-significantly increase in cHL	Cell survival signaling primarily for T-cells	Plasma membrane	Confirmed in HRS cells [45,46]
*CCL3*	Increased in cHL cases	Increased in cHL cases	Inflammation, cell survival signaling, and chemotaxis	Secreted	Confirmed in HRS cells [9]
*MCP-4*	Increased in cHL cases	Non-significantly increase in cHL	Chemotaxis and inflammation for monocytes and T-cells	Secreted	Confirmed in HRS cells [7]
*MMP12*	Increased in cHL cases	Increased in cHL cases	Modulating extracellular matrix. Produced by macrophages	Secreted, mainly extracellular matrix	No data available
*GZMB*	Increased in cHL cases	Non-significantly increase in cHL	Apoptosis/cytotoxic	Secreted	Confirmed in HRS cells and cytotoxic lymphocytes [47]

Functional and cellular annotation is based on a variety of public-access bioinformatic databases including UniProt (https://www.uniprot.org, accessed on 14 November 2021), Human Protein Atlas (http://www.proteinatlas.org, accessed on 14 November 2021), DisGeNET (http://geneontology.org, accessed on 14 November 2021), and cBioportal (https://www.cbioportal.org/, accessed on 14 November 2021). Cases with a significant congruent difference in tissue and plasma are marked in bold (*n* = 8). * HRS = Hodgkin Reed–Sternberg cells, cHL = classical Hodgkin lymphoma, TAM = tumor-associated macrophages.

## Data Availability

The data presented in this study are available upon reasonable request from the corresponding author. The raw data are not publicly available due to the fact of central ethical issues and privacy restrictions.

## Supplementary Materials

The following are available online at https://www.mdpi.com/article/10.3390/cancers14010009/s1, Table S1: Abbreviations for biomarkers in the Olink Immuno-Oncology panel; Table S2: Comparing proteome profiles in tissue; Table S3: Comparing proteome profiles in plasma; Table S4: Clinical correlations; Table S5: Correlations among cHL tissue distinguishing proteins.
