# Supplementary material for: Immune-Proteome Profiling in Classical Hodgkin Lymphoma Tumor Diagnostic Tissue
Source: Cancers (Basel). 2021 Dec 21;14(1):9. doi: 10.3390/cancers14010009 (PMC8750205; doi:10.3390/cancers14010009)
Supplement: Supplementary file 1 [file cancers-14-00009-s001.zip › Table_S1.pdf]

Table S1. Abbreviations for Biomarkers in the Olink Immuno-Oncology panel.

1. Adenosine Deaminase (ADA)
2. Adhesion G-protein coupled receptor G1 (ADGRG1)
3. Angiopoietin-1 (ANGPT1))
4. Angiopoietin-1 receptor (TIE2)
5. Angiopoietin-2 (ANGPT2)
6. Arginase-1 (ARG1)
7. C-C motif chemokine 17 (CCL17)
8. C-C motif chemokine 19 (CCL19)
9. C-C motif chemokine 20 (CCL20)
10. C-C motif chemokine 23 (CCL23)
11. C-C motif chemokine 3 (CCL3)
12. C-C motif chemokine 4 (CCL4)
13. C-X-C motif chemokine 1 (CXCL1)
14. C-X-C motif chemokine 10 (CXCL10)
15. C-X-C motif chemokine 11 (CXCL11)
16. C-X-C motif chemokine 13 (CXCL13)
17. C-X-C motif chemokine 5 (CXCL5)
18. C-X-C motif chemokine 9 (CXCL9)
19. Carbonic anhydrase IX (CAIX)
20. Caspase-8 (CASP-8)
21. CD27 antigen (CD27)
22. CD40 ligand (CD40-L)
23. CD40L receptor (CD40)
24. CD70 antigen (CD70)
25. CD83 antigen (CD83)
26. Cytotoxic and regulatory T-cell molecule (CRTAM)
27. Decorin (DCN)
28. Fas antigen ligand (FasL)
29. Fibroblast growth factor 2 (FGF2)
30. Fractalkine (CX3CL1)
31. Galectin-1 (Gal-1)
32. Galectin-9 (Gal-9)
33. Granzyme A (GZMA)
34. Granzyme B (GZMB)
35. Granzyme H (GZMH)
36. Heme oxygenase 1 (HO-1)
37. Hepatocyte growth factor (HGF)
38. ICOS ligand (ICOSLG)
39. Interferon gamma (IFN-gamma)
40. Interleukin-1 alpha (IL-1 alpha)
41. Interleukin-10 (IL10)
42. Interleukin-12 (IL-12)
43. Interleukin-12 receptor subunit beta-1 (IL12RB1)
44. Interleukin-13 (IL-13)
45. Interleukin-15 (IL15)
46. Interleukin-18 (IL-18)
47. Interleukin-2 (IL-2)
48. Interleukin-33 (IL-33)
49. Interleukin-4 (IL-4)
50. Interleukin-5 (IL5)
51. Interleukin-6 (IL6)
52. Interleukin-7 (IL-7)
53. Interleukin-8 (IL-8)
54. Killer cell immunoglobulin-like receptor 3DL1 (KIR3DL1)
55. Latency-associated peptide transforming growth factor beta-1 (LAP TGF-beta-1)
56. Lymphocyte activation gene 3 protein (LAG3)
57. Lysosome-associated membrane glycoprotein 3 (LAMP3)
58. Macrophage colony-stimulating factor 1 (CSF-1)
59. Matrix metalloproteinase-12 (MMP-12)
60. Matrix metalloproteinase-7 (MMP-7)
61. MHC class I polypeptide-related sequence A/B (MIC-A/B)
62. Monocyte chemotactic protein 1 (MCP-1)
63. Monocyte chemotactic protein 2 (MCP-2)
64. Monocyte chemotactic protein 3 (MCP-3)
65. Monocyte chemotactic protein 4 (MCP-4)
66. Mucin-16 (MUC-16)
67. Natural cytotoxicity triggering receptor 1 (NCR1)
68. Natural killer cell receptor 2B4 (CD244)
69. Natural killer cells antigen CD94 (KLRD1)
70. Nitric oxide synthase. endothelial (NOS3)
71. Placenta growth factor (PGF)
72. Platelet-derived growth factor subunit B (PDGF subunit B)
73. Pleiotrophin (PTN)
74. Pro-epidermal growth factor (EGF)
75. Programmed cell death 1 ligand 1 (PD-L1)
76. Programmed cell death 1 ligand 2 (PD-L2)
77. Programmed cell death protein 1 (PDCD1)
78. Stromal cell-derived factor 1 (CXCL12)
79. T-cell surface glycoprotein CD4 (CD4)
80. T-cell surface glycoprotein CD5 (CD5)
81. T-cell surface glycoprotein CD8 alpha chain (CD8A)
82. T-cell-specific surface glycoprotein CD28 (CD28)
83. TNF-related apoptosis-inducing ligand (TRAIL)
84. Tumor necrosis factor (Ligand) superfamily. member 12 (TWEAK)
85. Tumor necrosis factor (TNF)
86. Tumor necrosis factor ligand superfamily member 14 (TNFSF14)
87. Tumor necrosis factor receptor superfamily member 12A (TNFRSF12A)
88. Tumor necrosis factor receptor superfamily member 21 (TNFRSF21)
89. Tumor necrosis factor receptor superfamily member 4 (TNFRSF4)
90. Tumor necrosis factor receptor superfamily member 9 (TNFRSF9)
91. Vascular endothelial growth factor A (VEGF-A)
92. Vascular endothelial growth factor receptor 2 (VEGFR-2)
