# Supplementary material for: Immune-Proteome Profiling in Classical Hodgkin Lymphoma Tumor Diagnostic Tissue
Source: Cancers (Basel). 2021 Dec 21;14(1):9. doi: 10.3390/cancers14010009 (PMC8750205; doi:10.3390/cancers14010009)
Supplement: Supplementary file 1 [file cancers-14-00009-s001.zip › Table_S2.pdf]

Table S2. Comparing Proteome Profile in Tissue.

	<LOD freq. in cHL	<LOD freq. in Ctrl	<LOD freq. in all	Mean NPX Ctrl	Mean NPX HL	Mean NPX Diff.	T	P	Padj	Multi-variate: T	Multi-variate: P	Multi-variate: Padj	AUC	Pw	Pw(adj)	PS: P	PS: Padj
**LAG3**	0%	0%	0%	4.403	6.522	2.119	-7.776	<0.001	<0.001	7.854	<0.001	<0.001	0.925	<0.001	<0.001	<0.001	<0.001
**CCL17**	0%	0%	0%	7.219	11.019	3.800	-7.677	<0.001	<0.001	7.919	<0.001	<0.001	0.917	<0.001	<0.001	<0.001	<0.001
**IL6**	0%	7%	4%	4.142	7.021	2.879	-7.691	<0.001	<0.001	8.433	<0.001	<0.001	0.922	<0.001	<0.001	<0.001	<0.001
**IL13**	15%	87%	53%	3.284	5.666	2.382	-7.741	<0.001	<0.001	8.325	<0.001	<0.001	0.909	<0.001	<0.001	<0.001	<0.001
**CCL4**	0%	0%	0%	6.275	8.002	1.728	-6.066	<0.001	<0.001	6.630	<0.001	<0.001	0.878	<0.001	<0.001	<0.001	<0.001
**IFN-gamma**	0%	10%	5%	5.073	7.436	2.363	-6.166	<0.001	<0.001	6.427	<0.001	<0.001	0.893	<0.001	<0.001	<0.001	<0.001
**TIE2**	0%	0%	0%	3.770	3.124	-0.645	5.854	<0.001	<0.001	-5.736	<0.001	<0.001	0.870	<0.001	<0.001	<0.001	<0.001
**TNFRSF4**	0%	0%	0%	8.495	9.755	1.260	-5.196	<0.001	<0.001	5.186	<0.001	<0.001	0.835	<0.001	<0.001	<0.001	0.002
**PD-L1**	0%	0%	0%	7.268	8.362	1.094	-5.173	<0.001	0.001	5.573	<0.001	<0.001	0.854	<0.001	<0.001	<0.001	0.003
**MCP-1**	0%	0%	0%	8.566	9.551	0.985	-4.832	<0.001	0.001	4.655	<0.001	0.002	0.825	<0.001	0.001	<0.001	0.015
**MCP-2**	0%	0%	0%	4.930	6.618	1.688	-4.829	<0.001	0.001	4.815	<0.001	0.001	0.823	<0.001	0.001	<0.001	0.022
**IL7**	0%	0%	0%	3.644	3.059	-0.585	4.748	<0.001	0.001	-4.675	<0.001	0.002	0.833	<0.001	<0.001	<0.001	0.003
**CD70**	0%	0%	0%	3.213	4.012	0.799	-4.666	<0.001	0.003	4.890	<0.001	0.001	0.799	<0.001	0.005	<0.001	0.017
**CCL3**	0%	0%	0%	7.226	8.518	1.291	-4.541	<0.001	0.003	4.839	<0.001	0.001	0.805	<0.001	0.003	<0.001	0.029
**MCP-4**	0%	0%	0%	6.907	8.996	2.089	-4.447	<0.001	0.006	4.686	<0.001	0.002	0.821	<0.001	0.001	<0.001	0.029
**MMP12**	0%	0%	0%	7.687	9.578	1.891	-4.261	<0.001	0.007	4.249	<0.001	0.007	0.817	<0.001	0.001	<0.001	0.024
**GZMB**	0%	0%	0%	9.784	10.736	0.952	-3.621	0.001	0.049	3.673	0.001	0.042	0.762	0.001	0.040	0.001	0.071
ICOSLG	33%	23%	28%	2.890	2.674	-0.217	3.272	0.002	0.150	-3.056	0.004	0.256	0.708	0.007	0.457	0.007	0.461
PDCD1	0%	0%	0%	6.478	5.707	-0.771	3.231	0.002	0.156	-3.444	0.001	0.084	0.737	0.002	0.137	0.004	0.304
MCP-3	56%	70%	63%	3.052	3.570	0.518	-3.287	0.003	0.205	3.473	0.001	0.078	0.684	0.003	0.203	0.007	0.470
IL33	0%	0%	0%	5.413	4.678	-0.735	3.048	0.004	0.280	-2.990	0.004	0.300	0.711	0.006	0.405	0.006	0.396
GZMH	0%	0%	0%	10.763	11.599	0.836	-3.026	0.004	0.289	3.033	0.004	0.270	0.673	0.025	1.000	0.002	0.128
CXCL5	4%	3%	4%	6.870	5.737	-1.133	2.959	0.005	0.318	-2.912	0.005	0.367	0.722	0.004	0.308	0.012	0.831

	<LOD freq. in cHL	<LOD freq. in Ctrl	<LOD freq. in all	Mean NPX Ctrl	Mean NPX HL	Mean NPX Diff.	T	P	Padj	Multi-variate: T	Multi-variate: P	Multi-variate: Padj	AUC	Pw	Pw(adj)	PS: P	PS: Padj
LAMP3	0%	0%	0%	4.897	4.274	-0.624	2.869	0.006	0.404	-2.707	0.009	0.621	0.715	0.005	0.351	0.016	1.000
CCL19	0%	0%	0%	13.144	13.785	0.641	-2.784	0.007	0.508	2.849	0.006	0.430	0.716	0.005	0.338	0.004	0.275
VEGFR-2	0%	0%	0%	4.514	4.126	-0.388	2.772	0.008	0.510	-2.688	0.010	0.643	0.672	0.026	1.000	0.005	0.374
IL8	4%	7%	5%	4.178	4.822	0.644	-1.408	0.165	1.000	1.352	0.182	1.000	0.638	0.075	1.000	0.371	1.000
TNFRSF9	0%	0%	0%	9.956	9.881	-0.076	0.306	0.761	1.000	-0.335	0.739	1.000	0.490	0.905	1.000	0.855	1.000
CD40-L	15%	7%	11%	4.615	4.306	-0.309	2.035	0.047	1.000	-1.979	0.053	1.000	0.658	0.041	1.000	0.104	1.000
IL-1 alpha	96%	83%	89%	2.333	2.277	-0.056	1.062	0.294	1.000	-0.891	0.377	1.000	0.452	0.287	1.000	0.389	1.000
CD244	0%	0%	0%	6.261	6.458	0.197	-0.879	0.385	1.000	0.861	0.393	1.000	0.523	0.769	1.000	0.488	1.000
EGF	63%	60%	61%	2.102	1.897	-0.205	1.870	0.071	1.000	-1.634	0.108	1.000	0.442	0.397	1.000	0.224	1.000
ANGPT1	0%	0%	0%	2.166	2.097	-0.069	0.501	0.618	1.000	-0.421	0.676	1.000	0.540	0.617	1.000	0.810	1.000
PGF	0%	0%	0%	6.063	6.307	0.244	-0.999	0.322	1.000	0.961	0.341	1.000	0.574	0.344	1.000	0.236	1.000
ADGRG1	96%	93%	95%	3.034	3.004	-0.030	1.242	0.224	1.000	-1.049	0.299	1.000	0.467	0.339	1.000	0.224	1.000
CRTAM	11%	7%	9%	3.847	3.874	0.027	-0.143	0.887	1.000	0.129	0.898	1.000	0.509	0.917	1.000	0.902	1.000
CXCL11	0%	0%	0%	7.019	7.920	0.902	-2.109	0.040	1.000	2.187	0.033	1.000	0.651	0.052	1.000	0.075	1.000
TRAIL	0%	0%	0%	6.936	7.093	0.156	-0.818	0.418	1.000	0.899	0.373	1.000	0.553	0.500	1.000	0.609	1.000
FGF2	0%	0%	0%	5.618	6.009	0.391	-2.397	0.020	1.000	2.261	0.028	1.000	0.688	0.015	0.967	0.012	0.814
CXCL9	0%	0%	0%	9.764	10.694	0.930	-2.374	0.021	1.000	2.372	0.021	1.000	0.680	0.019	1.000	0.020	1.000
CD8A	0%	0%	0%	9.955	9.962	0.007	-0.020	0.984	1.000	0.177	0.860	1.000	0.498	0.981	1.000	0.725	1.000
CAIX	37%	67%	53%	1.904	2.334	0.430	-2.117	0.043	1.000	2.229	0.030	1.000	0.653	0.033	1.000	0.072	1.000
MUC-16	81%	80%	81%	2.280	2.265	-0.015	0.538	0.593	1.000	-0.464	0.644	1.000	0.488	0.826	1.000	0.611	1.000
ADA	0%	0%	0%	5.029	4.850	-0.179	1.058	0.295	1.000	-1.073	0.288	1.000	0.605	0.178	1.000	0.172	1.000
CD4	0%	0%	0%	9.255	9.035	-0.221	1.420	0.161	1.000	-1.309	0.196	1.000	0.653	0.048	1.000	0.338	1.000
NOS3	0%	0%	0%	7.788	7.576	-0.212	0.864	0.391	1.000	-0.775	0.442	1.000	0.590	0.249	1.000	0.276	1.000
IL2	48%	53%	51%	3.146	3.063	-0.083	1.292	0.203	1.000	-1.159	0.252	1.000	0.489	0.884	1.000	0.175	1.000
Gal-9	0%	0%	0%	10.551	10.412	-0.139	1.221	0.230	1.000	-1.291	0.202	1.000	0.417	0.290	1.000	0.187	1.000
CD40	0%	0%	0%	13.050	13.031	-0.019	0.329	0.743	1.000	-0.302	0.763	1.000	0.569	0.378	1.000	0.798	1.000
IL18	0%	0%	0%	10.014	10.021	0.008	-0.025	0.980	1.000	-0.144	0.886	1.000	0.522	0.781	1.000	0.887	1.000
KIR3DL1	74%	83%	79%	3.139	3.149	0.010	-0.177	0.860	1.000	0.259	0.796	1.000	0.538	0.494	1.000	0.864	1.000

	<LOD freq. in cHL	<LOD freq. in Ctrl	<LOD freq. in all	Mean NPX Ctrl	Mean NPX HL	Mean NPX Diff.	T	P	Padj	Multi-variate: T	Multi-variate: P	Multi-variate: Padj	AUC	Pw	Pw(adj)	PS: P	PS: Padj
LAP TGF-beta-1	0%	0%	0%	7.803	7.905	0.103	-0.568	0.573	1.000	0.654	0.516	1.000	0.562	0.432	1.000	0.445	1.000
CXCL1	0%	0%	0%	5.429	5.623	0.194	-0.635	0.528	1.000	0.723	0.473	1.000	0.558	0.460	1.000	0.738	1.000
TNFSF14	0%	0%	0%	6.046	6.087	0.041	-0.215	0.831	1.000	0.216	0.830	1.000	0.516	0.843	1.000	0.771	1.000
TWEAK	0%	0%	0%	7.979	7.715	-0.265	0.710	0.481	1.000	-0.632	0.530	1.000	0.530	0.709	1.000	0.844	1.000
PDGF subunit B	0%	0%	0%	3.898	3.978	0.080	-0.209	0.835	1.000	0.242	0.810	1.000	0.443	0.470	1.000	0.613	1.000
FASLG	0%	0%	0%	6.211	6.484	0.273	-1.272	0.210	1.000	1.287	0.204	1.000	0.563	0.423	1.000	0.224	1.000
CD28	15%	23%	19%	3.328	3.436	0.108	-1.232	0.223	1.000	1.246	0.218	1.000	0.620	0.122	1.000	0.542	1.000
IL15	41%	40%	40%	4.358	4.519	0.161	-1.853	0.072	1.000	1.929	0.059	1.000	0.575	0.317	1.000	0.199	1.000
Gal-1	0%	0%	0%	8.079	8.088	0.010	-0.201	0.841	1.000	0.142	0.888	1.000	0.586	0.269	1.000	0.741	1.000
CD27	0%	0%	0%	8.349	7.953	-0.396	2.303	0.026	1.000	-2.244	0.029	1.000	0.688	0.015	0.967	0.046	1.000
IL5	37%	37%	37%	2.726	2.673	-0.054	0.638	0.526	1.000	-0.582	0.563	1.000	0.520	0.799	1.000	0.473	1.000
HGF	0%	0%	0%	9.425	9.744	0.319	-0.633	0.530	1.000	0.675	0.503	1.000	0.567	0.395	1.000	0.439	1.000
GZMA	0%	0%	0%	12.681	12.704	0.023	-0.198	0.844	1.000	0.215	0.830	1.000	0.517	0.830	1.000	0.448	1.000
HO-1	0%	0%	0%	13.263	13.328	0.065	-0.363	0.718	1.000	0.277	0.783	1.000	0.596	0.217	1.000	0.834	1.000
CX3CL1	48%	53%	51%	1.921	1.875	-0.046	0.851	0.399	1.000	-0.699	0.488	1.000	0.491	0.911	1.000	0.252	1.000
CXCL10	0%	0%	0%	9.297	10.297	1.000	-2.029	0.048	1.000	2.068	0.043	1.000	0.633	0.086	1.000	0.143	1.000
IL10	78%	87%	82%	3.052	3.134	0.082	-1.420	0.166	1.000	1.454	0.152	1.000	0.551	0.329	1.000	0.247	1.000
TNFRSF12A	96%	87%	91%	1.976	1.940	-0.035	1.650	0.109	1.000	-1.446	0.154	1.000	0.451	0.198	1.000	0.106	1.000
CCL23	0%	0%	0%	4.613	5.300	0.687	-1.953	0.060	1.000	2.244	0.029	1.000	0.622	0.116	1.000	0.204	1.000
CD5	0%	0%	0%	11.854	11.636	-0.218	1.163	0.250	1.000	-1.081	0.285	1.000	0.609	0.163	1.000	0.393	1.000
MMP7	70%	50%	60%	3.226	3.299	0.073	-0.449	0.656	1.000	0.421	0.675	1.000	0.593	0.177	1.000	0.900	1.000
ARG1	78%	97%	88%	5.358	5.414	0.056	-0.905	0.372	1.000	1.050	0.298	1.000	0.510	0.843	1.000	0.331	1.000
NCR1	26%	3%	14%	3.105	2.902	-0.203	2.167	0.035	1.000	-2.168	0.035	1.000	0.691	0.014	0.919	0.055	1.000
DCN	0%	0%	0%	6.429	6.156	-0.273	1.381	0.176	1.000	-1.390	0.170	1.000	0.546	0.562	1.000	0.153	1.000
TNFRSF21	0%	0%	0%	2.197	1.887	-0.310	2.059	0.045	1.000	-2.044	0.046	1.000	0.657	0.043	1.000	0.083	1.000
MIC-A/B	19%	30%	25%	3.042	3.109	0.067	-0.590	0.558	1.000	0.672	0.504	1.000	0.561	0.430	1.000	0.715	1.000
ANGPT2	0%	0%	0%	5.085	5.394	0.310	-1.531	0.132	1.000	1.560	0.125	1.000	0.617	0.132	1.000	0.165	1.000
PTN	7%	13%	11%	4.641	4.740	0.099	-0.225	0.823	1.000	0.359	0.721	1.000	0.479	0.792	1.000	0.821	1.000

	<LOD freq. in cHL	<LOD freq. in Ctrl	<LOD freq. in all	Mean NPX Ctrl	Mean NPX HL	Mean NPX Diff.	T	P	Padj	Multi-variate: T	Multi-variate: P	Multi-variate: Padj	AUC	Pw	Pw(adj)	PS: P	PS: Padj
CXCL12	100%	87%	93%	2.253	2.219	-0.035	1.877	0.071	1.000	-1.752	0.086	1.000	0.433	0.054	1.000	0.070	1.000
CASP-8	0%	0%	0%	11.589	11.281	-0.308	1.300	0.202	1.000	-1.323	0.192	1.000	0.530	0.709	1.000	0.203	1.000
CXCL13	0%	0%	0%	12.036	12.468	0.432	-2.338	0.023	1.000	2.220	0.031	1.000	0.679	0.020	1.000	0.067	1.000
PD-L2	15%	33%	25%	2.757	2.790	0.033	-0.459	0.648	1.000	0.542	0.590	1.000	0.610	0.154	1.000	0.889	1.000
VEGFA	0%	0%	0%	4.348	4.729	0.381	-1.575	0.121	1.000	1.600	0.116	1.000	0.614	0.145	1.000	0.119	1.000
IL4	56%	37%	46%	2.679	2.363	-0.316	2.142	0.037	1.000	-2.302	0.025	1.000	0.656	0.034	1.000	0.032	1.000
IL12RB1	15%	3%	9%	3.720	4.024	0.304	-2.519	0.015	1.000	2.602	0.012	0.792	0.696	0.011	0.767	0.061	1.000
CCL20	19%	3%	11%	6.489	5.621	-0.868	1.831	0.073	1.000	-1.718	0.092	1.000	0.618	0.129	1.000	0.086	1.000
TNF	0%	0%	0%	5.321	5.384	0.064	-0.210	0.834	1.000	0.169	0.867	1.000	0.512	0.880	1.000	0.845	1.000
KLRD1	4%	0%	2%	3.040	3.229	0.189	-1.071	0.291	1.000	1.196	0.237	1.000	0.570	0.369	1.000	0.269	1.000
CD83	0%	0%	0%	4.766	4.568	-0.198	0.936	0.353	1.000	-0.891	0.377	1.000	0.581	0.298	1.000	0.578	1.000
IL12	4%	0%	2%	4.586	4.681	0.096	-0.360	0.720	1.000	0.372	0.712	1.000	0.535	0.663	1.000	0.620	1.000
CSF-1	0%	0%	0%	6.744	7.019	0.276	-1.978	0.053	1.000	2.029	0.048	1.000	0.647	0.058	1.000	0.094	1.000

Full Cohort (n=57)   cHLpatients, (n=27) versus controls (n=30). Bold proteins have significant differences between groups compared in both univariate and multivariate analysis adjusting for age and gender.

- Mean NPX Ctrl=Mean Normalized Protein eXpression (NPX) value in controls:
- Mean NPX cHL= Mean NPX value in patients with classical Hodgkin Lymphoma(cHL)
- Mean NPX diff.= Mean NPX cHL minus mean NPX Ctrl: One unit log2 NPX difference corresponds to a twofold difference in proteins concentration in the tissue
- T(t-value) and P(=p-value) Retrieved with Welch's t-test
- Padj=Adjusted P-value for multiple testing using. Method Benjamini-Hochberg's
- PS= Propensity scored matching. caliper=0.2. adjusting for gender and age. Comparing a homogenous group of 48.
- AUC=Area under curve. retrieved with Receiver Operating Curves (ROC)
- Multivariate p-values retrieved with linear regression and adjusting for age and sex.
- Lower CI= Lower 95% confident interval. Upper CI=Upper 95%confidens interval
- Pw= P-value retrieved with Wilcoxon rank-sum test
- < LOD freq..= Frequency of patients with values below the limit of detection (LOD)
