# Supplementary material for: Immune-Proteome Profiling in Classical Hodgkin Lymphoma Tumor Diagnostic Tissue
Source: Cancers (Basel). 2021 Dec 21;14(1):9. doi: 10.3390/cancers14010009 (PMC8750205; doi:10.3390/cancers14010009)
Supplement: Supplementary file 1 [file cancers-14-00009-s001.zip › Table_S3.pdf]

Table S3. Comparing Proteome Profile in Plasma.

	<LOD Freq	Mean NPX Ctrl	Mean NPX cHL	Mean NPX Diff.	T	P	Padj	Multi-variate: T	Multi-variate: P	Multi-variate: Padj	AUC	Pw	Pw(adj)	PS: P	PS: Padj
**IL7**	0%	4.482	6.157	1.675	-7.656	<0.001	<0.001	7.633	<0.001	<0.001	0.919	<0.001	<0.001	<0.001	<0.001
**IL6**	35%	3.159	2.007	2.007	-5.747	<0.001	<0.001	5.776	<0.001	<0.001	0.947	<0.001	<0.001	<0.001	0.001
**FGF2**	54%	1.779	2.900	1.121	-6.131	<0.001	<0.001	6.154	<0.001	<0.001	0.862	<0.001	<0.001	<0.001	0.001
**CD40**	0%	9.992	11.136	1.144	-7.340	<0.001	<0.001	7.414	<0.001	<0.001	0.920	<0.001	<0.001	<0.001	<0.001
**CXCL1**	0%	7.046	9.790	2.744	-10.663	<0.001	<0.001	11.252	<0.001	<0.001	0.976	<0.001	<0.001	<0.001	<0.001
**CCL19**	0%	10.757	12.846	2.089	-7.847	<0.001	<0.001	7.828	<0.001	<0.001	0.954	<0.001	<0.001	<0.001	<0.001
**PD.L1**	0%	5.966	6.781	0.816	-5.819	<0.001	<0.001	5.804	<0.001	<0.001	0.883	<0.001	<0.001	<0.001	<0.001
**CXCL5**	0%	8.709	11.995	3.286	-10.220	<0.001	<0.001	10.629	<0.001	<0.001	0.963	<0.001	<0.001	<0.001	<0.001
**IL10**	0%	3.389	4.976	1.587	-7.629	<0.001	<0.001	7.646	<0.001	<0.001	0.973	<0.001	<0.001	<0.001	<0.001
**MMP7**	0%	8.794	10.884	2.090	-11.707	<0.001	<0.001	12.046	<0.001	<0.001	0.994	<0.001	<0.001	<0.001	<0.001
**ARG1**	8%	6.177	4.643	-1.534	8.456	<0.001	<0.001	-8.823	<0.001	<0.001	0.963	<0.001	<0.001	<0.001	<0.001
**TNFRSF4**	0%	6.400	7.383	0.983	-5.957	<0.001	<0.001	6.303	<0.001	<0.001	0.886	<0.001	<0.001	<0.001	<0.001
**CCL17**	0%	9.508	11.737	2.228	-7.025	<0.001	<0.001	7.544	<0.001	<0.001	0.912	<0.001	<0.001	<0.001	<0.001
**CXCL13**	0%	8.386	10.864	2.478	-8.989	<0.001	<0.001	9.002	<0.001	<0.001	0.946	<0.001	<0.001	<0.001	<0.001
**TNFRSF9**	0%	6.453	7.417	0.964	-5.208	<0.001	0.001	6.065	<0.001	<0.001	0.858	<0.001	<0.001	<0.001	0.001
**ICOSLG**	0%	6.170	5.836	-0.334	4.950	<0.001	0.001	-4.944	<0.001	0.001	0.838	<0.001	0.001	<0.001	0.007
**LAG3**	0%	4.667	5.835	1.168	-5.085	<0.001	0.001	5.230	<0.001	<0.001	0.856	<0.001	<0.001	<0.001	0.001
**CD244**	0%	6.919	7.455	0.536	-4.595	<0.001	0.003	4.565	<0.001	0.002	0.819	<0.001	0.002	<0.001	0.008
**CCL3**	0%	5.547	6.361	0.814	-4.624	<0.001	0.003	5.104	<0.001	<0.001	0.811	<0.001	0.004	<0.001	0.002
**CASP.8**	0%	6.336	5.322	-1.013	4.576	<0.001	0.003	-4.666	<0.001	0.002	0.806	<0.001	0.005	0.001	0.037
**EGF**	0%	7.149	8.879	1.729	-4.381	<0.001	0.004	4.354	<0.001	0.004	0.802	<0.001	0.006	<0.001	0.007
**CXCL9**	0%	7.053	8.744	1.691	-4.450	<0.001	0.004	5.320	<0.001	<0.001	0.803	<0.001	0.006	<0.001	0.001
**MMP12**	0%	6.783	7.832	1.049	-4.380	<0.001	0.004	4.519	<0.001	0.003	0.806	<0.001	0.005	<0.001	0.001

	<LOD Freq	Mean NPX Ctrl	Mean NPX cHL	Mean NPX Diff.	T	P	Padj	Multi-variate: T	Multi-variate: P	Multi-variate: Padj	AUC	Pw	Pw(adj)	PS: P	PS: Padj
**TNF**	0%	4.540	5.475	0.934	-4.476	<0.001	0.005	4.584	<0.001	0.002	0.822	<0.001	0.002	<0.001	0.003
**CXCL11**	0%	8.024	9.456	1.432	-4.254	<0.001	0.006	4.536	<0.001	0.003	0.795	<0.001	0.009	<0.001	0.002
**IL13**	72%	2.879	3.466	0.586	-4.590	<0.001	0.007	4.702	<0.001	0.002	0.770	<0.001	0.004	<0.001	0.019
**PDCD1**	0%	5.148	5.886	0.738	-4.231	<0.001	0.010	4.639	<0.001	0.002	0.816	<0.001	0.003	<0.001	0.004
**CSF.1**	0%	10.656	10.947	0.291	-4.025	<0.001	0.014	3.992	<0.001	0.014	0.809	<0.001	0.004	<0.001	0.007
**TRAIL**	0%	8.187	8.499	0.312	-3.677	0.001	0.043	3.691	0.001	0.035	0.785	<0.001	0.016	0.002	0.119
**NOS3**	71%	3.054	3.410	0.356	-3.788	0.001	0.048	3.894	<0.001	0.019	0.778	<0.001	0.003	0.001	0.059
IL15	0%	5.178	5.432	0.255	-3.173	0.003	0.159	3.146	0.003	0.160	0.729	0.004	0.215	0.004	0.231
CXCL10	0%	9.564	10.611	1.047	-3.162	0.003	0.186	3.718	0.001	0.033	0.712	0.007	0.404	0.001	0.039
CD27	0%	8.049	8.430	0.381	-3.099	0.003	0.205	3.354	0.002	0.094	0.712	0.007	0.404	0.003	0.201
DCN	0%	4.475	4.196	-0.279	3.084	0.004	0.217	-3.329	0.002	0.100	0.776	<0.001	0.025	0.004	0.235
IL8	0%	4.486	5.229	0.743	-3.030	0.004	0.224	3.213	0.002	0.137	0.725	0.004	0.253	0.002	0.113
CCL23	0%	10.784	11.318	0.534	-3.090	0.004	0.224	3.191	0.002	0.143	0.756	0.001	0.066	0.003	0.194
TNFSF14	0%	4.747	5.353	0.605	-2.961	0.005	0.260	2.946	0.005	0.272	0.719	0.006	0.315	0.004	0.231
TWEAK	0%	8.828	8.548	-0.280	2.866	0.006	0.342	-2.827	0.007	0.353	0.701	0.012	0.567	0.064	1.000
CD40.L	0%	6.283	7.443	1.160	-2.815	0.007	0.375	2.886	0.006	0.313	0.708	0.009	0.460	0.004	0.231
MCP.3	52%	2.543	2.774	0.231	-2.642	0.012	0.648	2.678	0.010	0.513	0.665	0.025	1.000	0.018	0.873
CRTAM	0%	5.686	6.233	0.547	-2.602	0.012	0.648	2.875	0.006	0.316	0.705	0.010	0.503	0.019	0.898
Gal.9	0%	7.823	8.115	0.292	-2.535	0.015	0.763	2.950	0.005	0.272	0.715	0.007	0.368	0.011	0.545
KLRD1	0%	6.493	6.943	0.450	-2.486	0.016	0.816	2.647	0.011	0.533	0.698	0.013	0.618	0.012	0.587
CD5	0%	6.280	6.570	0.291	-2.499	0.017	0.849	2.663	0.010	0.523	0.695	0.014	0.671	0.018	0.878
PD.L2	0%	3.470	3.665	0.195	-2.443	0.018	0.878	2.423	0.019	0.899	0.675	0.028	1.000	0.008	0.438
TIE2	0%	8.143	8.037	-0.106	1.392	0.170	1.000	-1.410	0.165	1.000	0.627	0.116	1.000	0.310	1.000
IL.1.alpha	98%	1.843	1.847	0.004	-1.000	0.327	1.000	2.135	0.038	1.000	0.519	0.326	1.000	0.073	1.000
ANGPT1	0%	8.444	8.470	0.025	-0.076	0.939	1.000	0.067	0.947	1.000	0.499	0.993	1.000	0.553	1.000
PGF	0%	8.661	8.809	0.148	-1.464	0.150	1.000	1.492	0.142	1.000	0.604	0.199	1.000	0.134	1.000
ADGRG1	75%	2.481	2.683	0.203	-1.496	0.147	1.000	1.546	0.128	1.000	0.501	1.000	1.000	0.244	1.000

	<LOD Freq	Mean NPX Ctrl	Mean NPX cHL	Mean NPX Diff.	T	P	Padj	Multi-variate: T	Multi-variate: P	Multi-variate: Padj	AUC	Pw	Pw(adj)	PS: P	PS: Padj
MCP.1	0%	10.796	10.999	0.203	-1.111	0.272	1.000	1.250	0.217	1.000	0.600	0.218	1.000	0.315	1.000
MCP.4	0%	10.503	10.796	0.293	-1.132	0.263	1.000	1.297	0.201	1.000	0.595	0.239	1.000	0.336	1.000
CD8A	0%	10.200	10.559	0.359	-1.880	0.066	1.000	1.842	0.072	1.000	0.631	0.104	1.000	0.058	1.000
CAIX	0%	4.740	4.326	-0.414	2.359	0.022	1.000	-2.580	0.013	0.620	0.704	0.010	0.520	0.046	1.000
MUC.16	6%	3.177	3.507	0.330	-1.390	0.173	1.000	1.291	0.203	1.000	0.603	0.208	1.000	0.053	1.000
ADA	0%	6.019	6.351	0.332	-2.216	0.033	1.000	2.277	0.027	1.000	0.647	0.068	1.000	0.008	0.427
CD4	0%	3.888	4.088	0.200	-2.059	0.046	1.000	2.216	0.031	1.000	0.679	0.025	1.000	0.039	1.000
IL2	98%	2.840	2.833	-0.006	1.041	0.307	1.000	-1.019	0.313	1.000	0.463	0.169	1.000	0.310	1.000
VEGFR.2	0%	8.878	8.888	0.010	-0.122	0.904	1.000	0.100	0.921	1.000	0.469	0.704	1.000	0.380	1.000
IL18	0%	10.397	10.027	-0.369	1.664	0.102	1.000	-1.693	0.097	1.000	0.637	0.089	1.000	0.454	1.000
GZMH	0%	5.220	5.177	-0.043	0.155	0.877	1.000	-0.123	0.902	1.000	0.504	0.965	1.000	0.792	1.000
KIR3DL1	46%	2.983	2.841	-0.142	0.929	0.357	1.000	-0.908	0.368	1.000	0.596	0.216	1.000	0.516	1.000
LAP.TGF.beta.1	0%	9.421	9.732	0.311	-1.506	0.138	1.000	1.500	0.140	1.000	0.621	0.134	1.000	0.087	1.000
IL33	100%	1.839	1.839	<0.001	1.000	0.327	1.000	-1.006	0.319	1.000	0.481	0.345	1.000	0.329	1.000
PDGF.subunit.B	0%	10.199	10.490	0.291	-1.210	0.232	1.000	1.195	0.238	1.000	0.631	0.104	1.000	0.070	1.000
FASLG	0%	7.483	7.242	-0.240	1.616	0.113	1.000	-1.593	0.118	1.000	0.632	0.100	1.000	0.282	1.000
CD28	94%	2.733	2.787	0.054	-1.206	0.239	1.000	1.262	0.213	1.000	0.558	0.076	1.000	0.241	1.000
MCP.2	0%	7.772	8.137	0.364	-1.589	0.118	1.000	1.721	0.092	1.000	0.624	0.124	1.000	0.052	1.000
CCL4	0%	6.261	6.505	0.244	-1.400	0.168	1.000	1.508	0.138	1.000	0.625	0.120	1.000	0.092	1.000
Gal.1	0%	7.121	7.153	0.032	-0.366	0.716	1.000	0.390	0.698	1.000	0.561	0.453	1.000	0.837	1.000
IL5	35%	2.709	2.642	-0.067	0.536	0.595	1.000	-0.424	0.673	1.000	0.486	0.872	1.000	0.852	1.000
HGF	0%	9.322	9.618	0.296	-2.004	0.051	1.000	2.036	0.047	1.000	0.640	0.083	1.000	0.023	1.000
GZMA	0%	7.702	7.735	0.034	-0.211	0.834	1.000	0.234	0.816	1.000	0.462	0.640	1.000	0.563	1.000
HO.1	0%	12.904	12.832	-0.072	0.594	0.556	1.000	-0.581	0.564	1.000	0.561	0.453	1.000	0.473	1.000
CX3CL1	0%	4.047	3.957	-0.090	0.893	0.377	1.000	-0.927	0.359	1.000	0.584	0.301	1.000	0.504	1.000
CD70	0%	3.601	3.831	0.230	-1.531	0.134	1.000	1.716	0.093	1.000	0.591	0.261	1.000	0.136	1.000
TNFRSF12A	0%	5.203	4.996	-0.208	1.411	0.165	1.000	-1.585	0.119	1.000	0.613	0.164	1.000	0.228	1.000

	<LOD Freq	Mean NPX Ctrl	Mean NPX cHL	Mean NPX Diff.	T	P	Padj	Multi-variate: T	Multi-variate: P	Multi-variate: Padj	AUC	Pw	Pw(adj)	PS: P	PS: Padj
NCR1	0%	4.242	4.319	0.077	-0.708	0.482	1.000	0.780	0.439	1.000	0.548	0.554	1.000	0.433	1.000
TNFRSF21	0%	7.903	7.790	-0.113	1.771	0.083	1.000	-1.774	0.082	1.000	0.631	0.104	1.000	0.100	1.000
MIC.A.B	5%	5.416	5.903	0.487	-1.589	0.119	1.000	1.626	0.110	1.000	0.584	0.301	1.000	0.070	1.000
ANGPT2	0%	6.249	6.651	0.403	-2.248	0.029	1.000	2.357	0.022	1.000	0.661	0.045	1.000	0.071	1.000
PTN	66%	2.564	2.664	0.100	-1.102	0.277	1.000	1.368	0.178	1.000	0.527	0.709	1.000	0.273	1.000
CXCL12	66%	2.000	2.080	0.080	-1.686	0.100	1.000	1.680	0.099	1.000	0.557	0.432	1.000	0.067	1.000
IFN.gamma	2%	6.615	7.469	0.855	-2.291	0.028	1.000	2.378	0.021	0.982	0.682	0.022	1.000	0.023	1.000
LAMP3	0%	5.197	5.300	0.102	-0.597	0.553	1.000	0.735	0.466	1.000	0.541	0.615	1.000	0.372	1.000
VEGFA	0%	9.859	10.202	0.343	-2.263	0.028	1.000	2.253	0.029	1.000	0.645	0.071	1.000	0.034	1.000
IL4	86%	2.010	2.124	0.114	-0.774	0.446	1.000	0.734	0.466	1.000	0.454	0.409	1.000	0.354	1.000
IL12RB1	68%	2.646	2.789	0.143	-2.041	0.047	1.000	2.182	0.034	1.000	0.707	0.002	0.115	0.007	0.365
CCL20	0%	6.525	7.248	0.723	-2.068	0.044	1.000	2.037	0.047	1.000	0.735	0.003	0.171	0.047	1.000
GZMB	0%	4.572	4.240	-0.333	1.572	0.122	1.000	-1.579	0.121	1.000	0.581	0.318	1.000	0.539	1.000
CD83	0%	3.218	3.447	0.229	-1.836	0.075	1.000	2.131	0.038	1.000	0.662	0.043	1.000	0.032	1.000
IL12	0%	7.508	7.777	0.269	-1.117	0.271	1.000	1.167	0.249	1.000	0.631	0.104	1.000	0.181	1.000

Full Cohort (n=53); cHL(n=26) versus controls(n=27). Bold proteins are significant differences between groups compared in a univariate and multivariate analysis adjusting for age and gender(shown first). Protein CXCL10 marked with italic was significant after adjusting for age and gender.

- Mean NPX Ctrl=Mean Normalized Protein eXpression (NPX) value in controls:
- Mean NPX cHL= Mean NPX value in patients with classical Hodgkin Lymphoma(cHL)
- Mean NPX diff.= Mean NPX cHL minus mean NPX Ctrl: One unit log2 NPX difference corresponds to a two-fold difference in proteins concentration in the tissue
- T(t-value) and P(=p-value) Retrieved with Welch's t-test
- Padj.=Adjusted P-value for multiple testing using. Method Benjamini-Hochberg's
- PS= Propensity scored matching. caliper=0.2. adjusting for gender and age. Comparing a homogenous group of 42.
- AUC=Area under curve. retrieved with Receiver Operating Curves (ROC)
- Multivariate p-values retrieved with linear regression and adjusting for age and sex.
- Lower CI= Lower 95% confident interval. Upper CI=Upper 95%confidens interval
- Pw= P-value retrieved with Wilcoxon rank-sum test
- < LOD freq.= Frequency of patients with values below the limit of detection (LOD)
