# Supplementary material for: Immune-Proteome Profiling in Classical Hodgkin Lymphoma Tumor Diagnostic Tissue
Source: Cancers (Basel). 2021 Dec 21;14(1):9. doi: 10.3390/cancers14010009 (PMC8750205; doi:10.3390/cancers14010009)
Supplement: Supplementary file 1 [file cancers-14-00009-s001.zip › Table_S4.pdf]

Table S4. Clinical Correlations.

	EFS		OS		Advance stage		CR		EBV		NS		Male sex		Age	
	HR	p	HR	p	p	Diff.	p	Diff.	p	Diff	p	Diff	p	Diff	p	Corr.
**IL8**	1.062	0.761	1.310	0.238	0.388	0.640	0.554	-0.503	0.032	-1.266	0.013	1.538	0.322	-0.955	0.882	0.030
**TNFRSF9**	0.670	0.314	0.487	0.223	0.922	0.037	0.352	0.457	0.754	0.118	0.466	-0.247	0.209	0.523	0.360	0.183
**TIE2**	0.184	0.277	<0.001	0.263	0.687	-0.060	0.992	0.001	0.900	-0.023	0.915	-0.017	0.334	-0.143	0.619	-0.100
**MCP.3**	1.449	0.487	1.610	0.510	0.229	0.404	0.437	-0.281	0.152	-0.394	0.030	0.591	0.077	-0.777	0.726	0.071
**CD40.L**	0.188	0.089	<0.001	0.997	0.112	-0.361	0.403	0.252	0.906	-0.033	0.410	0.192	0.911	-0.026	0.985	-0.004
**IL.1.alpha**	<0.001	0.998	0.587	1.000	0.318	-0.048	0.355	-0.077	0.314	-0.036	0.316	0.043	0.365	0.036	0.227	-0.240
**CD244**	0.988	0.979	0.802	0.785	0.454	0.296	0.906	-0.049	0.027	1.257	0.195	-0.539	0.157	0.507	0.330	0.195
**EGF**	104.865	0.154	0.007	0.709	0.169	-0.065	0.088	-0.103	0.802	0.015	0.387	-0.052	0.841	0.012	0.426	-0.160
**ANGPT1**	1.323	0.847	<0.001	0.153	0.915	-0.017	0.073	-0.266	0.146	-0.247	0.045	0.283	0.172	-0.246	0.515	-0.131
**IL7**	0.257	0.119	<0.001	0.995	0.955	0.011	0.190	0.270	0.317	0.238	0.540	-0.130	0.889	0.030	0.714	0.074
**PGF**	0.599	0.327	0.704	0.660	0.559	-0.224	0.557	0.249	0.512	-0.287	0.578	0.203	0.257	-0.463	0.581	0.111
**IL6**	1.296	0.304	1.086	0.825	0.111	-1.140	0.852	-0.173	0.307	0.937	0.257	0.944	0.603	0.405	0.002	-0.568
**ADGRG1**	<0.001	0.999	<0.001	1.000	0.334	-0.001	0.333	0.001	0.330	-0.001	0.332	0.001	0.331	0.001	0.562	-0.117
**MCP.1**	1.893	0.274	1.422	0.706	0.655	0.126	0.519	-0.187	0.137	0.361	0.095	0.487	0.557	-0.204	0.676	0.084
**CRTAM**	0.893	0.832	0.870	0.856	0.682	0.120	0.846	0.069	0.008	1.150	0.084	-0.543	0.249	0.301	0.207	0.251
**CXCL11**	0.939	0.776	1.039	0.901	0.042	1.452	0.961	-0.042	0.008	2.413	0.201	-1.040	0.205	0.854	0.699	0.078
**MCP.4**	0.974	0.917	0.382	0.204	0.454	-0.688	0.666	0.443	0.235	-0.812	0.017	2.011	0.431	-0.779	0.069	-0.355
**TRAIL**	0.856	0.784	0.347	0.386	0.128	0.511	0.747	0.113	0.004	1.019	0.228	-0.393	0.115	0.590	0.751	0.064
**FGF2**	0.287	0.156	0.331	0.493	0.656	0.106	0.616	0.123	0.594	0.177	0.704	-0.097	0.603	-0.109	0.067	0.358
**CXCL9**	1.157	0.613	5.192	0.202	0.263	0.667	0.934	-0.058	0.001	1.651	0.134	-0.883	0.425	0.500	0.356	0.185
**CD8A**	0.425	0.046	0.011	0.478	0.318	-0.468	0.324	0.594	0.021	1.275	0.204	-0.572	0.552	-0.231	0.477	0.143
**CAIX**	1.941	0.092	0.206	0.675	0.869	0.069	0.848	-0.092	0.034	-0.599	0.029	0.719	0.567	-0.270	0.629	-0.097
**MUC.16**	0.254	0.740	<0.001	1.000	0.710	0.012	0.282	0.033	0.438	0.038	0.722	-0.013	0.812	-0.009	0.971	0.007

	EFS		OS		Advance stage		CR		EBV		NS		Male sex		Age	
	HR	p	HR	p	p	Diff.	p	Diff.	p	Diff	p	Diff	p	Diff	p	Corr.
**ADA**	0.374	0.109	0.023	0.290	0.925	0.027	0.237	0.389	0.101	0.571	0.764	-0.087	0.331	0.241	0.588	0.109
**CD4**	0.304	0.082	<0.001	0.995	0.999	<0.001	0.317	0.262	0.922	0.023	0.282	0.253	0.505	0.144	0.827	-0.044
**NOS3**	0.449	0.087	<0.001	0.996	0.780	0.097	0.225	0.494	0.783	0.105	0.863	0.060	0.543	-0.198	0.333	0.194
**IL2**	0.020	0.401	<0.001	0.993	0.406	-0.053	0.723	0.026	0.252	0.103	0.724	-0.024	0.958	0.004	0.393	-0.171
**Gal.9**	1.764	0.692	3.250	0.771	0.691	0.078	0.393	-0.160	0.798	0.049	0.161	-0.245	0.364	0.274	0.802	0.051
**VEGFR.2**	0.494	0.438	0.428	0.624	0.524	0.134	0.840	0.040	0.799	0.067	0.617	-0.104	0.880	-0.036	0.398	0.170
**CD40**	0.036	0.196	0.008	0.279	0.724	0.024	0.405	0.074	0.068	0.130	0.227	-0.078	0.085	0.126	0.790	0.054
**IL18**	0.896	0.827	5.251	0.212	0.071	0.865	0.618	-0.318	0.311	0.493	0.146	-0.727	0.028	1.028	0.058	0.369
**GZMH**	0.999	0.999	2.283	0.496	0.946	0.020	0.824	-0.058	0.316	0.302	0.841	-0.057	0.801	0.085	0.875	0.032
**KIR3DL1**	<0.001	0.996	0.826	1.000	0.327	-0.079	0.230	0.093	0.343	0.155	0.974	-0.003	0.948	0.005	0.754	-0.063
**LAP.TGF.beta.1**	0.622	0.489	0.107	0.273	0.565	0.175	0.385	0.232	0.909	0.049	0.391	0.259	0.197	-0.425	0.698	0.078
**CXCL1**	0.656	0.341	1.104	0.932	0.881	0.074	0.772	0.109	0.045	-0.909	0.026	1.039	0.973	0.020	0.245	-0.232
**TNFSF14**	0.374	0.051	<0.001	0.994	0.133	-0.468	0.163	0.535	0.358	0.381	0.621	0.175	0.857	0.052	0.996	0.001
**IL33**	0.639	0.281	0.125	0.202	0.700	0.162	0.137	0.611	0.515	0.341	0.998	-0.001	0.828	-0.096	0.492	0.138
**TWEAK**	0.623	0.244	0.627	0.595	0.955	-0.030	0.449	0.370	0.700	-0.264	0.236	0.602	0.171	-0.804	0.775	0.058
**PDGF.subunit.B**	0.961	0.906	1.145	0.764	0.489	0.353	0.746	-0.175	0.513	0.451	0.966	-0.023	0.522	-0.325	0.633	0.096
**PDCD1**	0.499	0.314	0.001	0.224	0.927	-0.029	0.347	0.305	0.268	0.398	0.114	-0.485	0.561	0.177	0.078	0.345
**FASLG**	0.805	0.681	0.421	0.472	0.131	0.523	0.722	-0.134	0.045	1.110	0.181	-0.503	0.034	0.649	0.188	0.261
**CD28**	0.090	0.141	0.015	0.231	0.846	-0.024	0.690	0.062	0.552	0.090	0.909	0.015	0.817	-0.028	0.856	-0.037
**CCL19**	0.292	0.009	<0.001	0.274	0.874	-0.057	0.007	1.194	0.455	0.256	0.484	0.239	0.760	-0.111	0.983	-0.004
**MCP.2**	1.501	0.208	1.060	0.872	0.042	1.281	0.521	-0.413	0.267	0.833	0.544	0.429	0.328	0.583	0.901	-0.025
**CCL4**	1.223	0.557	0.956	0.931	0.108	0.692	0.395	-0.424	0.015	1.231	0.499	-0.296	0.003	1.065	0.632	0.096
**IL15**	1.377	0.730	0.326	0.564	0.300	0.174	0.773	0.047	0.034	0.422	0.552	-0.096	0.041	0.277	0.703	0.077
**Gal.1**	0.207	0.441	0.022	0.375	0.637	0.038	0.483	0.055	0.715	-0.043	0.225	0.113	0.699	-0.032	0.675	0.084
**PD.L1**	1.072	0.870	0.572	0.444	0.207	0.494	0.684	-0.177	0.020	1.129	0.849	-0.082	0.005	0.894	0.567	-0.115

	EFS		OS		Advance stage		CR		EBV		NS		Male sex		Age	
	**HR**	**p**	**HR**	**p**	**p**	**Diff.**	**p**	**Diff.**	**p**	**Diff**	**p**	**Diff**	**p**	**Diff**	**p**	**Corr.**
**CD27**	0.351	0.020	0.046	0.305	0.739	-0.099	0.161	0.556	0.044	0.526	0.636	-0.151	0.913	0.030	0.861	-0.035
**CXCL5**	0.505	0.054	0.427	0.286	0.753	-0.171	0.957	0.028	0.386	-0.638	0.335	0.590	0.520	-0.332	0.205	-0.252
**IL5**	0.017	0.239	0.005	0.594	0.918	-0.011	0.616	0.059	0.544	0.100	0.930	-0.010	0.111	-0.150	0.960	-0.010
**HGF**	1.025	0.932	0.694	0.633	0.377	0.617	0.874	-0.111	0.203	-1.243	0.217	0.992	0.160	-1.176	0.862	0.035
**GZMA**	1.614	0.732	2.941	0.687	0.553	-0.094	0.763	0.043	0.623	-0.114	0.985	-0.003	0.584	-0.074	0.721	0.072
**HO.1**	6.387	0.205	0.421	0.733	0.199	0.387	0.250	-0.322	0.131	0.425	0.358	-0.261	0.301	0.482	0.350	0.187
**CX3CL1**	3.244	0.654	<0.001	0.999	0.671	0.027	0.704	-0.026	0.614	-0.034	0.329	0.058	0.837	-0.015	0.624	-0.099
**CXCL10**	1.023	0.917	1.099	0.781	0.027	1.844	0.872	-0.155	0.006	2.515	0.183	-1.123	0.107	1.247	0.425	0.160
**CD70**	0.940	0.913	0.804	0.813	0.867	0.050	0.522	-0.233	0.690	0.136	0.709	0.114	0.042	0.579	0.920	0.020
**IL10**	<0.001	0.982	<0.001	0.990	0.514	0.078	0.176	0.135	0.306	0.155	0.470	0.070	0.647	-0.067	0.950	0.013
**TNFRSF12A**	<0.001	0.999	0.010	1.000	0.334	-0.005	0.347	-0.009	0.356	0.011	0.343	-0.008	0.331	0.004	0.279	-0.216
**CCL23**	1.299	0.423	0.281	0.180	0.927	0.065	0.936	0.067	0.400	-0.431	0.042	1.268	0.659	-0.342	0.091	-0.332
**CD5**	0.522	0.098	0.217	0.323	0.459	-0.238	0.308	0.433	0.189	0.333	0.486	0.251	0.826	0.064	0.479	-0.142
**CCL3**	1.227	0.598	0.891	0.844	0.113	0.690	0.708	-0.178	0.011	1.191	0.236	-0.483	0.019	0.846	0.371	0.179
**MMP7**	1.221	0.712	1.832	0.376	0.079	0.611	0.231	-0.474	0.936	-0.029	0.390	-0.314	0.984	0.007	0.120	0.306
**ARG1**	0.349	0.715	NA	0.342	0.210	-0.137	0.453	0.065	0.137	-0.122	0.138	0.143	0.474	-0.114	0.350	-0.187
**NCR1**	0.085	0.143	<0.001	0.323	0.483	-0.104	0.165	0.215	0.074	0.432	0.269	-0.167	0.369	-0.133	0.962	0.010
**DCN**	0.822	0.689	0.334	0.307	0.628	0.177	0.344	-0.309	0.007	-1.511	0.005	1.244	0.369	-0.306	0.604	-0.104
**TNFRSF21**	0.361	0.118	0.173	0.161	0.654	-0.121	0.305	0.266	0.038	-0.692	0.099	0.523	0.284	-0.271	0.866	0.034
**TNFRSF4**	0.789	0.613	0.180	0.207	0.725	-0.129	0.771	0.138	0.881	-0.055	0.024	0.752	0.182	0.470	0.279	-0.216
**MIC.A.B**	0.041	0.089	0.001	0.268	0.814	-0.039	0.817	-0.045	0.390	0.182	0.770	0.049	0.643	0.088	0.246	-0.231
**CCL17**	1.129	0.529	0.908	0.694	0.244	-1.020	0.483	0.765	0.162	-1.685	0.029	2.048	0.226	-0.993	0.006	-0.515
**ANGPT2**	0.459	0.191	0.085	0.234	0.881	-0.046	0.060	0.549	0.799	0.104	0.607	0.157	0.714	-0.128	0.987	0.003
**PTN**	0.914	0.757	0.940	0.902	0.681	-0.280	0.850	0.136	0.193	-0.898	0.101	1.059	0.295	-0.838	0.495	-0.137
**CXCL12**	*NA*	*NA*	*NA*	*NA*	*NA*	*NA*	*NA*	*NA*	*NA*	*NA*	*NA*	*NA*	*NA*	*NA*	*NA*	*NA*

	EFS		OS		Advance stage		CR		EBV		NS		Male sex		Age	
	HR	p	HR	p	p	Diff.	p	Diff.	p	Diff	p	Diff	p	Diff	p	Corr.
**IFN.gamma**	1.051	0.826	1.175	0.599	0.168	0.937	0.817	-0.174	0.011	2.234	0.289	-0.830	0.193	0.889	0.640	0.094
**LAMP3**	0.507	0.300	0.734	0.760	0.504	-0.191	0.189	0.428	0.417	0.252	0.813	0.065	0.579	0.191	0.260	-0.224
**CASP.8**	0.689	0.257	<0.001	0.395	0.788	0.114	0.692	0.179	0.348	0.446	0.898	0.058	0.459	0.400	0.959	0.010
**ICOSLG**	<0.001	0.076	<0.001	0.998	0.399	-0.058	0.494	0.055	0.768	0.026	0.794	-0.020	0.410	-0.063	0.116	-0.310
**MMP12**	0.957	0.853	0.626	0.217	0.273	-0.853	0.096	1.616	0.113	1.125	0.446	0.641	0.918	0.076	0.896	0.026
**CXCL13**	0.517	0.213	0.531	0.441	0.915	0.027	0.151	0.412	0.234	-0.406	0.420	0.229	0.440	-0.223	0.768	0.060
**PD.L2**	0.035	0.164	<0.001	0.993	0.824	0.018	0.417	0.079	0.778	-0.023	0.565	0.048	0.431	-0.064	0.434	-0.157
**VEGFA**	0.742	0.593	0.716	0.737	0.750	-0.116	0.659	-0.170	0.902	-0.052	0.534	0.225	0.205	-0.518	0.957	0.011
**IL4**	0.023	0.360	<0.001	0.998	0.313	-0.160	0.265	0.173	0.769	-0.041	0.139	-0.341	0.297	-0.261	0.357	0.184
**LAG3**	0.915	0.821	0.743	0.686	0.104	0.697	0.463	0.348	0.924	0.046	0.422	0.360	0.834	0.103	0.709	0.075
**IL12RB1**	0.303	0.158	0.081	0.191	0.331	0.200	0.875	0.040	0.138	0.435	0.887	-0.033	0.315	0.191	0.776	0.057
**IL13**	1.045	0.871	0.886	0.808	0.102	-1.076	0.080	1.220	0.610	0.329	0.107	0.965	0.666	-0.274	0.056	-0.372
**CCL20**	0.557	0.099	<0.001	0.996	0.946	0.045	0.643	0.322	0.470	-0.638	0.206	0.970	0.386	-0.646	0.147	-0.287
**TNF**	0.301	0.020	0.183	0.098	0.155	-0.577	0.209	0.614	0.444	-0.303	0.685	-0.168	0.979	0.013	0.876	0.032
**KLRD1**	0.845	0.800	0.182	0.272	0.966	-0.013	0.351	0.297	0.098	0.832	0.866	-0.050	0.182	0.364	0.974	-0.007
**GZMB**	1.477	0.394	1.262	0.719	0.318	0.343	0.766	-0.113	0.014	0.722	0.993	-0.003	0.332	0.458	0.326	-0.197
**CD83**	0.677	0.480	0.290	0.247	0.513	-0.182	0.062	0.625	0.801	-0.070	0.788	-0.071	0.823	0.072	0.762	0.061
**IL12**	0.457	0.214	2.728	0.409	0.196	-0.540	0.247	0.489	0.734	0.190	0.906	0.048	0.312	-0.449	0.390	0.172
**CSF.1**	0.692	0.637	0.122	0.242	0.914	0.023	0.807	-0.059	0.498	0.213	0.343	0.230	0.187	0.278	0.995	0.001

Correlations between patient tissues NPX values and different clinicopathological features. Number of events for EFS; n=8. and for OS; n=3: Hazard Ratio (=HR) with associated Walds-test for p values(=p) was retrieved with Cox Regression. According to Ann Arbor (Dichotomous), all survival analysis was adjusted for advanced stage(IIB-IVB) according to Ann Arbour (Dichotomous) and Age as a continuous variable. The investigated proteins were treated as a continuous variable in Cox regression. Pearson correlation with associated correlation coefficient(=corr) used when comparing two continuous variables. Welch's two-sample test was used for comparing a continuous variable with a dichotomic clinical variable. Diff= Difference of the mean in classical Hodgkin lymphoma (cHL) patient group minus the control group. EFS= Event-free survival OS= Overall survival. CR= Complete remission assessed with radiology after first-line treatment. EBV= Epstein Barr virus assessed with EBER. NS=Nodular sclerosis subtype of cHL. Proteins with high LOD frequency would automatically generate non-significant outcomes since values below LOD were replaced with LOD. or yield NA.
