# Supplementary material for: Immune-Proteome Profiling in Classical Hodgkin Lymphoma Tumor Diagnostic Tissue
Source: Cancers (Basel). 2021 Dec 21;14(1):9. doi: 10.3390/cancers14010009 (PMC8750205; doi:10.3390/cancers14010009)
Supplement: Supplementary file 1 [file cancers-14-00009-s001.zip › Table_S5.pdf]

Table S5. Correlations Between cHL Tissue Distinguishing Proteins.

	**IL6**	**PD.L1**	**CCL3**	**CCL17**	**LAG3**	**IL13**	**MCP.1**	**MCP.4**	**MCP.2**	**CCL4**	**IFN. gamma**	**TIE2**	**TNFRSF4**	**MMP12**	**IL7**	**CD70**	**GZMB**
**IL6**	NA	0.377	0.144	0.568	0.166	0.374	0.111	0.105	0.128	0.247	0.122	0.354	0.497	0.230	0.369	0.170	0.604
**PD.L1**	0.052	NA	**0.785**	-0.201	0.344	-0.014	0.339	0.012	0.662	**0.821**	0.632	0.069	0.367	0.319	0.339	0.228	0.605
**CCL3**	0.474	<0.001	NA	-0.407	0.391	-0.347	0.242	-0.354	0.557	0.886	0.650	-0.063	0.189	0.200	0.192	0.106	0.459
**CCL17**	0.002	0.314	0.035	NA	0.066	0.635	-0.087	0.414	-0.178	-0.324	-0.398	0.150	0.348	0.144	0.049	-0.086	0.114
**LAG3**	0.407	0.079	0.044	0.745	NA	-0.121	0.040	-0.078	0.240	0.453	0.496	0.193	0.318	0.026	0.238	0.198	0.533
**IL13**	0.054	0.946	0.076	<0.001	0.548	NA	0.111	0.602	-0.103	-0.175	-0.094	-0.031	0.279	0.489	-0.030	0.134	0.058
**MCP.1**	0.582	0.084	0.224	0.666	0.844	0.583	NA	0.245	0.596	0.328	0.234	-0.104	0.123	0.187	-0.053	0.090	0.181
**MCP.4**	0.603	0.953	0.070	0.032	0.699	0.001	0.218	NA	0.326	-0.280	-0.158	0.015	-0.029	0.243	-0.115	-0.223	-0.034
**MCP.2**	0.526	<0.001	0.003	0.374	0.228	0.609	0.001	0.097	NA	0.502	0.536	-0.150	-0.068	0.036	-0.086	-0.090	0.403
**CCL4**	0.215	<0.001	<0.001	0.099	0.018	0.384	0.095	0.158	0.008	NA	0.701	0.040	0.411	0.174	0.251	0.450	0.641
**IFN.gamma**	0.544	<0.001	<0.001	0.040	0.009	0.641	0.241	0.430	0.004	<0.001	NA	-0.052	-0.042	0.046	0.047	0.224	0.642
**TIE2**	0.070	0.731	0.755	0.455	0.335	0.880	0.607	0.940	0.457	0.844	0.795	NA	0.229	0.153	0.734	0.155	0.302
**TNFRSF4**	0.008	0.059	0.344	0.075	0.106	0.159	0.541	0.885	0.735	0.033	0.836	0.251	NA	0.380	0.391	0.663	0.271
**MMP12**	0.249	0.105	0.318	0.475	0.898	0.010	0.349	0.221	0.860	0.384	0.821	0.445	0.051	NA	0.324	0.117	0.099
**IL7**	0.058	0.084	0.338	0.809	0.233	0.881	0.793	0.569	0.668	0.206	0.814	<0.001	0.044	0.099	NA	0.153	0.352
**CD70**	0.396	0.253	0.600	0.668	0.323	0.504	0.655	0.263	0.655	0.018	0.260	0.439	<0.001	0.562	0.446	NA	0.269
**GZMB**	0.001	0.001	0.016	0.573	0.004	0.773	0.367	0.867	0.037	<0.001	<0.001	0.126	0.171	0.623	0.072	0.174	NA

NA= Not applicable, Grey area= Correlation coefficient, White area, = p-values, All retrieved with person correlation method.
